# Genetic Organization of the *aprX-lipA2* Operon Affects the Proteolytic Potential of *Pseudomonas* Species in Milk

**DOI:** 10.3389/fmicb.2020.01190

**Published:** 2020-06-10

**Authors:** Christopher Maier, Christopher Huptas, Mario von Neubeck, Siegfried Scherer, Mareike Wenning, Genia Lücking

**Affiliations:** ^1^ZIEL Institute for Food and Health, Wissenschaftszentrum Weihenstephan, Technische Universität München, Freising, Germany; ^2^Lehrstuhl für Mikrobielle Ökologie, Wissenschaftszentrum Weihenstephan, Technische Universität München, Freising, Germany; ^3^Bavarian Health and Food Safety Authority (LGL), Oberschleißheim, Germany

**Keywords:** *aprX-lipA2* operon, AprX peptidase, genus *Pseudomonas*, proteolytic potential, milk spoilage

## Abstract

Psychrotolerant *Pseudomonas* species are a main cause of proteolytic spoilage of ultra-high temperature (UHT) milk products due to the secretion of the heat-resistant metallopeptidase AprX, which is encoded by the first gene of the *aprX-lipA2* operon. While the proteolytic property has been characterized for many different *Pseudomonas* isolates, the underlying *aprX-lipA2* gene organization was only described for a few strains so far. In this study, the phylogenomic analysis of 185 *Pseudomonas* type strains revealed that the presence of *aprX* is strongly associated to a monophylum composed of 81 species, of which 83% carried the *aprX* locus. Furthermore, almost all type strains of known milk-relevant species were shown to be members of the three monophyletic groups *P. fluorescens*, *P. gessardii*, and *P. fragi*. In total, 22 different types of *aprX-lipA2* genetic organizations were identified in the genus, whereby 31% of the species tested carried the type 1 operon structure consisting of eight genes (*aprXIDEF prtAB lipA2*). Other genetic structures differed from type 1 mainly in the presence and location of genes coding for two lipases (*lipA1* and *lipA2*) and putative autotransporters (*prtA* and *prtB*). The peptidase activity of 129 strains, as determined on skim milk agar and in UHT-milk, correlated largely with different *aprX-lipA2* gene compositions. Particularly, isolates harboring the type 1 operon were highly proteolytic, while strains with other operon types, especially ones lacking *prtA* and *prtB*, exhibited significantly lower peptidase activities. In conclusion, the phylogenomic position and the *aprX-lipA2* gene organization specify the proteolytic potential of *Pseudomonas* isolates. In addition, however, an interplay of several environmental factors and intrinsic traits influences production and activity of AprX, leading to strain-specific proteolytic phenotypes.

## Introduction

Psychrotolerant bacteria, predominant in cold stored raw milk, are known to secrete heat-resistant enzymes, which partly even withstand ultra-high temperature processing (von Neubeck et al., 2015; Glück et al., 2016). Continuous enzyme activity during storage can cause product defects like off-flavors and textural flaws by decomposition of milk components, primary casein (Stoeckel et al., 2016). As a consequence, the quality of ultra-high temperature treated (UHT) products decreases and premature spoilage may occur, presenting considerable economic challenges for the milk industry (McKellar, 1981; Sørhaug and Stepaniak, 1997; Stoeckel et al., 2016). Especially, UHT products intended for long-distance transport, e.g., export to Asia, require prolonged shelf life to ensure consistent product stability and quality.

Representatives of the genera *Pseudomonas*, *Microbacterium*, *Acinetobacter*, and *Lactococcus* are particularly abundant in cold stored raw milk. Among them, pseudomonads were shown to be the main cause of proteolytic activity (Baur et al., 2015a; von Neubeck et al., 2015). Currently, the genus *Pseudomonas* comprises 188 species (March 2019), which makes it one of the largest bacterial genera known so far. *Pseudomonas* species are ubiquitous, gram-stain negative, non-spore forming, aerobic rods (Palleroni, 2015). After frequent reclassifications, the genus is presently divided into 21 monophyletic groups (Gomila et al., 2015; Peix et al., 2018). With respect to spoilage potential, previous studies revealed that milk-associated *Pseudomonas* strains differ strongly in their proteolytic properties, even if they are taxonomically closely related (Baur et al., 2015a). Apart from the cultivation temperature, other extrinsic and intrinsic factors such as the composition of the cultivation medium, iron content, quorum sensing, and phase variation were shown to influence peptidase activities of *Pseudomonas* members in a species‐ and partially strain-specific manner (Nicodeme et al., 2005; Maunsell et al., 2006; Liu et al., 2007; Alves et al., 2018). However, the complex regulatory processes behind these variations are not yet fully understood.

So far, only one extracellular peptidase has been characterized in *Pseudomonas*, namely the metallopeptidase AprX (Liao and McCallus, 1998; Woods et al., 2001). The nomenclature of AprX in literature is partly misleading as AprX was also entitled AprA in *P. aeruginosa* (Guzzo et al., 1991). However, in the context of proteolytic, milk-associated species, AprA and AprX are identical, whereas in *P. aeruginosa aprX* and *aprA* encode for two different proteins (Duong et al., 2001). The caseinolytic endopeptidase AprX belongs to the serralysin family and holds a Zn^2+^ ion and Ca^2+^ ions for stability and functionality (Schokker and van Boekel, 1997). It has a size of 45–50 kDa (Marchand et al., 2009), shows the highest substrate turnover at 37–45°C and is functional from slightly acidic to alkaline pH (Dufour et al., 2008; Marchand et al., 2009; Martins et al., 2015; Matéos et al., 2015). The corresponding *aprX* gene is located in the *aprX-lipA2* operon, consisting of several genes controlled by a single promotor upstream of the *aprX* gene. In *P. fluorescens* B52, the operon includes, besides *aprX*, genes encoding for a peptidase inhibitor (AprI), a type I secretion system (AprDEF), two putative autotransporter homologs (PrtAB), and a lipase (LipA2) (Woods et al., 2001). So far, the *aprX-lipA2* gene cluster is known to comprise up to nine different genes in total (Ma et al., 2003). Strain-specific deviations in the organization of the operon, for example, a missing *prtA* gene in *P. brassicacearum* NFM421, loss of *prtAB* in *P. fluorescens* SIK W1, an additional lipase gene in *P. fluorescens* Pf0-1, and a completely different operon structure in *P. aeruginosa* PAO-1 have been mentioned in the literature (Duong et al., 2001; Ma et al., 2003). However, the *aprX-lipA2* operon structure has only been described for very few *Pseudomonas* species up to now.

To close this gap, the aims of this study were an extensive analysis of the *aprX* gene distribution in the whole genus *Pseudomonas* as well as the clarification of the existing *aprX-lipA2* genetic organizations and their possible influence on peptidase production. Toward this end, a phylogenomic analysis of 185 type strains based on 92 bacterial core genes was conducted and the strain set screened for *aprX*-positive candidates. Besides, 87 *Pseudomonas* isolates, mainly from raw milk, were fully sequenced and the *aprX-lipA2* operon organization of all strains analyzed. Moreover, the proteolytic activity of selected strains with different genetic constitutions was determined in order to find potential correlations between peptidase production and the underlying operon structure.

## Materials and Methods

### Bacterial Strains and Growth Conditions

*Pseudomonas* strains used for sequencing and/or proteolytic profiling (Supplementary Tables S1 and S2) were grown aerobically on tryptic soy agar (TSA, Carl Roth GmbH) for 24 h at 30°C. For overnight cultures, 4 ml tryptic soy broth (TSB, Merck Millipore KGaA) were inoculated with material from one colony and incubated for 16 h at 30°C and 150 rpm.

### DNA Extraction

Genomic DNA (gDNA) was extracted from overnight cultures using the QIAamp® DNA Mini Kit (Qiagen). In contrast to the manufacturer’s instructions, samples were treated with Proteinase K for 4 h at 56°C. RNA digestion was performed for 30 min at 70°C using 10 μl of RNase A at a concentration of 10 mg/ml (Thermo Scientific). Final elution of gDNA from spin columns was carried out twice with 100 μl of sterile deionized water each. DNA concentrations were measured using a Qubit 2.0 Fluorometer (Invitrogen) and Qubit dsDNA HS Assay Kits (Invitrogen) in compliance with the manufacturer’s instructions.

### Whole-Genome Sequencing, Read Quality Control, and *de novo* Assembly

All 87 *Pseudomonas* strains sequenced *de novo* are summarized in Supplementary Table S1. For each strain, at least one sequencing library was prepared according to a modified version of the Illumina TruSeq DNA PCR-free Sample Preparation procedure (Huptas et al., 2016). Libraries were sequenced with the Illumina MiSeq System. Almost all sequencing runs were conducted using MiSeq Reagent v3 Kits (600-cycle) or v2 Kits (500-cycle). Library pools were demultiplexed with the on-board MiSeq Reporter Software. After visual inspection of raw read quality using FastQC v0.10.1^1^, reads were trimmed and filtered with the NGS QC Toolkit v2.2.3 (Patel and Jain, 2012). Reads were cut 10 nts from 5′ end and at least 1 nt from 3′ end. Low quality and adapter contaminated reads as well as those losing their counterpart during filtering were rejected. Finally, repeated visual analysis (FastQC) ensured that all remaining reads were of high quality. Supplementary Table S1 shows the read lengths and sequencing depths achieved for each individual sample. Genome reconstruction was performed with SPAdes v2.5.1 (Bankevich et al., 2012) using the assembler’s in-build functionalities for read error (Nikolenko et al., 2013) and contig mismatch correction. In any case, k-mers applied were 21, 33, 55, 77, 99, and 127. Contigs with less than 500 nts were removed from draft genome assemblies. For each sequenced strain, detailed assembly statistics are listed in Supplementary Table S1.

### Public Genome Data

Next to the 87 strains sequenced and assembled *de novo* (Supplementary Table S1), 184 *Pseudomonas* genome assemblies (Supplementary Table S3) were obtained from the National Center for Biotechnology Information (NCBI).

### Phylogenomic Tree Reconstruction and Monophyletic Group Assignment

**Figure 1:**
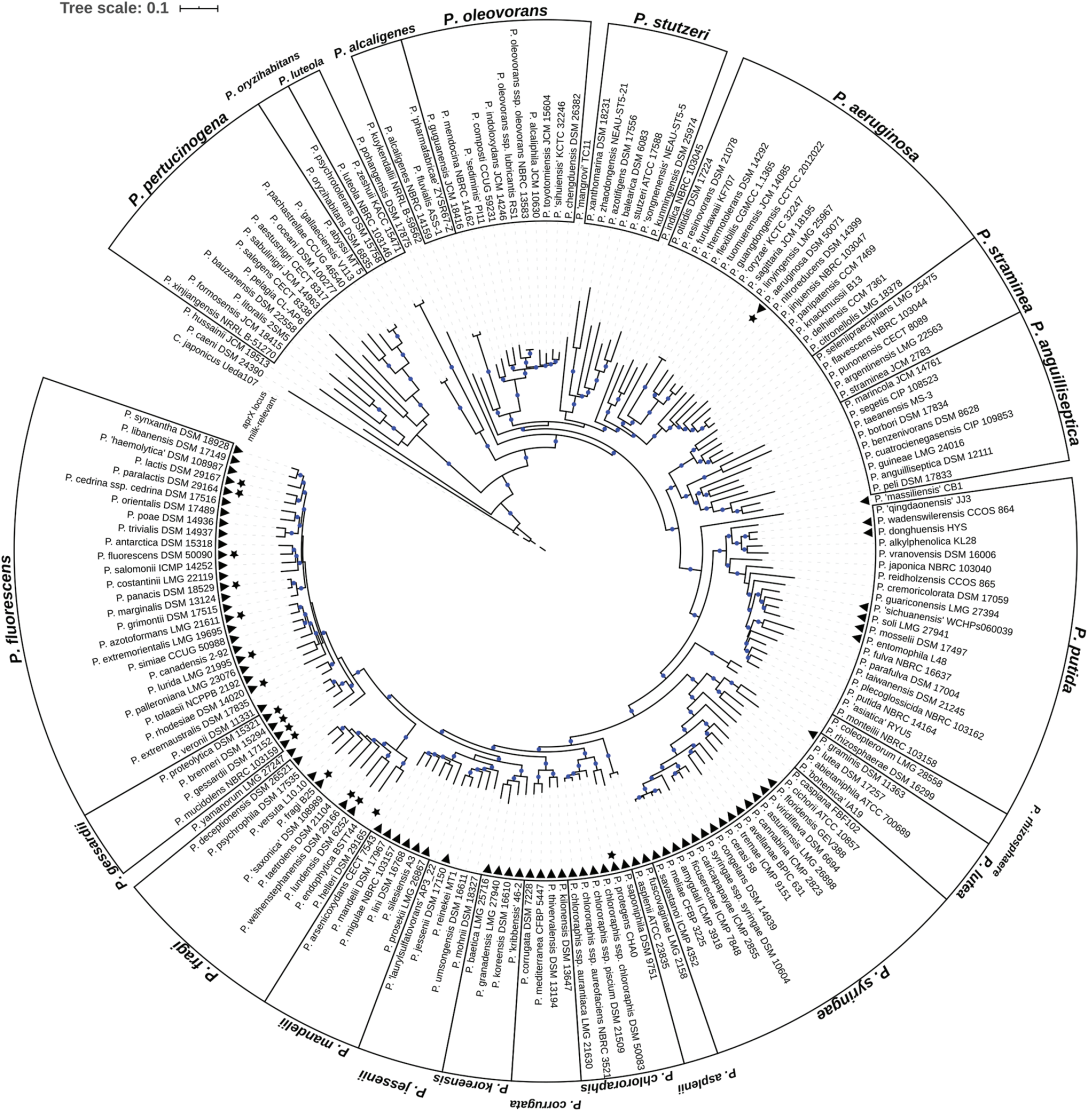
Phylogenomic tree of the genus *Pseudomonas*. Shown is the rooted maximum likelihood phylogenomy of the genus *Pseudomonas* comprising 185 type strains. The tree is based on a multiple sequence alignment composed of 92 universal bacterial core genes (69,704 alignment positions). Evolutionary distances were estimated using the GTR+G+I model. Branches with high bootstrap support (≥70%) are marked with blue circles. In total, 200 bootstrap replicates were calculated. *Cellvibrio japonicus* Ueda107^T^ (RefSeq ID NC_010995.1) is used as outgroup. For reasons of clarity the length of the outgroup branch was downscaled to 0.05 substitutions per site (dashed branch). Black triangles highlight type strains containing the *aprX* gene. Black stars refer to type strains of species classified as milk-relevant in the past. The genus is separated into 22 distinct monophyletic groups and 8 singletons. Group names are shown at the outer-most border.

**Figure 2:**
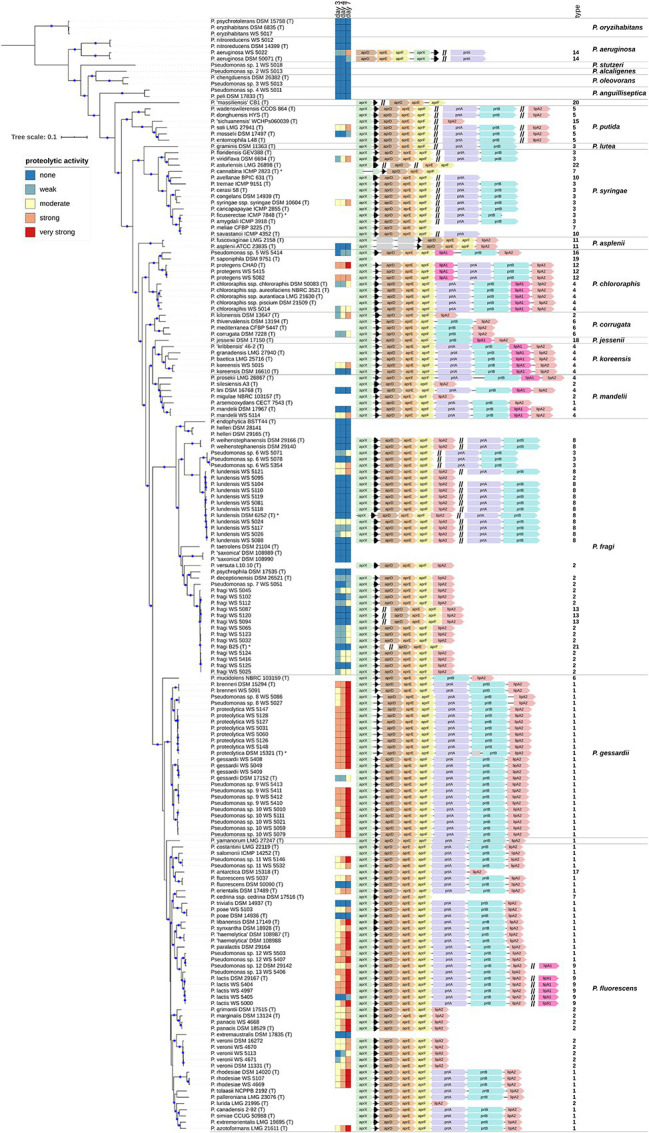
(Continued)FIGURE 2Proteolytic activity and *aprX-lipA2* genetic organizations of selected *Pseudomonas* strains. The rooted phylogenomy shows 178 *Pseudomonas* strains for which an agar diffusion assay was performed and/or the *aprX* gene was found. The maximum-likelihood method and the GTR+G+I model were applied to reconstruct the tree based on 92 concatenated universal bacterial core genes (75,078 alignment positions). Branches containing blue circles are of high bootstrap support (≥70% of 200 replicates). The monophylum represented by strains *P. psychrotolerans* DSM 15758^T^, *P. oryzihabitans* DSM 6835^T^, and *P. oryzihabitans* WS 5017 was used to root the tree (outgroup). Strain-specific proteolytic activity after 3, 4, and 7 days is visualized as heatmap illustrating non (dark blue), weak (light blue), moderate (yellow), strong (light red) and very strong (dark red) activities. *AprX-lipA2* genetic organizations are shown at single-nucleotide resolution next to the heatmap and in a strain-wise manner. Coding sequences not belonging to one of the genes *aprX* (green), *aprI* (black), *aprD* (brown), *aprE* (orange), *aprF* (yellow), *prtA* (magenta), *prtB* (cyan), *lipA1* (pink), and *lipA2* (antique pink) are colored gray. Individual genes or gene clusters of the *aprX-lipA2* operon being present at distinct genomic locations are separated by a double slash (//). In such cases, the different portions of genetic information do not necessarily have to lie on the same strand (be encoded in the same direction). Strains containing partial gene sequences are marked with an asterisk (*). In this context, left-open gene sequences indicate that the 5'-portion of the coding sequence is missing, whereas right-open gene sequences highlight cases lacking the 3'-part. Each *aprX-lipA2* genetic organization is followed by its type number. To provide a more structured overview the phylogenomy is divided into 18 monophyletic groups and two singletons (*P. massiliensis* CB1^T^ and *P. yamanorum* LMG 27247^T^). Group names are listed on the right-hand of the figure.

The UBCG pipeline v3.0 (Na et al., 2018) was applied to extract and align 92 universal bacterial core genes from genome sequence data. In general, standard parameter settings were applied with the exception of the filter cutoff that was set to zero. On the basis of the UBCG multiple sequence alignments, maximum likelihood phylogenomic trees were calculated using the MEGA X Software v10.0.5 (Kumar et al., 2018). To model DNA evolution, the General-Time-Reversible model incorporating rate heterogeneity (five discrete gamma categories) and a proportion of invariant sites (GTR+G+I) was chosen. Positions containing gaps in the multiple sequence alignments were not considered. All remaining parameters were kept at their default settings. In total, 200 bootstrap replicates were computed for each tree to infer branch confidence values. Bacterial strains present in both phylogenomies (Figures 1, 2) were separated into distinct clades according to a classification scheme already used to highlight the intrageneric structure of the genus *Pseudomonas* based on multi-gene phylogenies (Mulet et al., 2010; Gomila et al., 2015; Peix et al., 2018). In more detail, the positioning of each strain within the phylogenomy was used to assign it to one of the 21 monophyletic groups defined previously. In cases in which topological constrains (length and order of branches) prohibited the allocation to a known group, strains were kept as singletons (no group membership) or clustered together to form a new group. New groups were named after the first species described in that group. Both phylogenomies were visualized using the Interactive Tree Of Life (iTOL) online tool v5.3 (Letunic and Bork, 2019).

### Species Delineation and Naming of Strains

Species affiliation of non-type strains was determined using the Microbial Species Identifier (MiSi) software v1 (Varghese et al., 2015). First, each non-type strain was compared to all type strains and assigned to the particular nomenspecies, to which it shared the highest genome-wide average nucleotide identity (gANI), provided the observed gANI value was not less than the threshold for species demarcation (96.5%). Non-type strains not assignable in that way $\left(max\left(gANI\right)<96.5\%\right)$ were analyzed in a subsequent all-against-all comparison. Finally, non-type strains sharing gANI values above the species cutoff were clustered together. Based on this analysis, our strain collection contains 12 potentially new genomospecies. Non-type strains were named in accordance to the species they were assigned to during species delineation.

### Gene Prediction and Screening of *aprX-lipA2* Operon Genes

Prodigal v2.6 (Hyatt et al., 2010) was applied to predict the protein coding potential of each strain (Supplementary Tables S1 and S3) preventing genes to run off contig ends and forcing Shine-Dalgarno motif scans. Predicted gene sequences as well as corresponding protein translations were used for further analysis. A reference protein sequence of each of the nine known *aprX-lipA2* operon genes (GenBank IDs AGL85002.1 to AGL85010.1) was used to search for homologous sequences within the predicted protein coding content of each strain/genome investigated. For each strain and *aprX-lipA2* reference protein the best BLASTp v2.2.25+ (Camacho et al., 2009) hit was saved. Subsequently, best hits were part of manual curation taking alignment statistics (bit-score, e-value, percent identity, aligned fraction, etc.) and gene neighborhood into account to judge whether a best hit corresponds to an orthologous protein. If necessary, genomic loci of best hits were analyzed in detail to uncover single-nucleotide polymorphism and small insertion/deletion mutations. Multiple sequence alignments of genomic loci and gene sequences were calculated online using Clustal Omega v1.2.4 (Sievers and Higgins, 2014).

### Statistics

All statistical analyses were performed with R v3.6.1 (R Core Team, 2019). To test for nonrandom associations Fisher’s exact test (Fisher.test function of stats package) was used. One-way analysis of variance (aov function of stats package) in combination with Tukey’s multiple comparison test [Tukey_hsd function of rstatix package (Kassambara, 2019b)] was applied to test for significant differences in mean proteolytic activity (agar diffusion assay at days 3, 4, and 7) at species level of the most abundant *aprX-lipA2* genetic organizations (Figure 4 and Supplementary Figure S1). Associated boxplot representations were created using functionalities of the R packages ggplot2 (Wickham, 2016), ggpubr (Kassambara, 2019a), ggthemes (Arnold, 2019), cowplot (Wilke, 2019), grid, and gridExtra (Auguie, 2017). To meet test assumptions, normality of data and homogeneity of variance were checked with the Shapiro-Wilk (Shapiro.test function of stats package) and Levene’s test [LeveneTest function of car package (Fox and Weisberg, 2019)], respectively (Supplementary Table S4). In general, strains showing no proteolytic activity until day 7 (zero values) were omitted from statistical analyses to avoid a distortion of the test statistics.

**Figure 3:**
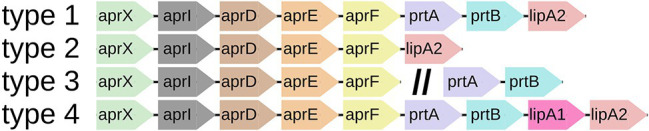
Most abundant *aprX-lipA2* genetic organizations. Schematic representation of the four most abundant *aprX-lipA2* genetic organizations (type 1–4) in *Pseudomonas*, as determined by number of species per type. The double slash (//) indicates the presence of particular gene clusters at distinct genomic locations.

### Measurement of Proteolytic Activity

Supplementary Table S2 lists 129 *Pseudomonas* strains for which agar diffusion assays or azocasein assays were performed. The strain set comprises 72 isolates from raw milk or semi-finished milk products, four isolates from water, two from food environment, one from a cow, one from soil, and three with unknown origin [Weihenstephan (WS) strain collection, TUM, Freising; DSMZ, Braunschweig], and 46 *Pseudomonas* type strains of diverse origins including raw milk (DSMZ, Braunschweig). For the agar diffusion assay, proteolytic activity of *Pseudomonas* strains was determined on skim milk agar [2× nutrient agar with 10% (w/v) skim milk powder; pH 6.8]. After suspending one loop of cell material in 100 μl sterile deionized water, the suspension was firmly vortexed and 15 μl were spotted on skim milk agar. After cultivation for 7 days at 12°C, clearing zones around the colonies indicated the proteolytic hydrolysis of casein. The extent of enzymatic activity Δr was assessed by subtracting the radius of the colony $r_{colony}$ from the radius of the clearing zone $r_{zone}$ in cm ($\Delta r=r_{zone}-r_{colony}$). Thereby all tested strains were categorized into non (Δr = 0 cm), weak (0 cm < Δr ≤ 0.2 cm), moderate (0.2 cm < Δr ≤ 0.5 cm), strong (0.5 cm < Δr < 1 cm), and very strong Δr ≥ 1 cm) peptidase producers. The azocasein assay was used to quantify the extracellular proteolytic activity in liquid cultures (Sørhaug and Stepaniak, 1997). Therefore, an overnight culture was used to inoculate 50 ml UHT milk (1.5% fat) with approximately 10^3^–10^4^ cfu and the culture was shaken at 150 rpm at 12°C for 4 days. Bacterial cultures were harvested (14,000 rpm, 12°C), the supernatants carefully sterile-filtered (0.22 μm; Berrytec GmbH) and frozen at −20°C until use. In parallel, cell counts were determined for each sample on TSA. Before measurement, the supernatants were diluted 10^−1^ and 10^−2^ in Ringer solution (Merck KGaA) and the azocasein assay was performed as previously described (Baur et al., 2015b) with slight modifications. In brief, the diluted supernatants and a freshly prepared azocasein-solution containing 0.5% (w/v) azocasein (Sigma-Aldrich), 50 mM MOPS buffer (pH 6.7), and 1 mM CaCl_2_ were preincubated separately at 40°C for 5 min. Then, 100 μl azocasein-solution was added to 100 μl of diluted supernatant and samples were incubated at 40°C for 1 h while shaking (600 rpm). The enzymatic reaction was stopped by adding 20 μl 2 M TCA to the sample. After centrifugation (14,000 rpm, 5 min), 150 μl of each sample was transferred into a microtiter plate containing 50 μl 1 M NaOH. Absorbance was measured at 450 nm in a plate reader (Victor3, PerkinElmer Inc.) and only absorption values below 0.59 were stated as linear and taken into account. Proteolytic activity (Sørhaug and Stepaniak, 1997) was defined as difference between absorption of blank and sample value at 450 nm per hour and ml enzyme solution $(\mathrm{EPA}=\frac{\Delta A}{\mathrm{h x ml}}).$ EPA values were normalized to the logarithm of 10^9^ cells and the according strains were categorized into low (0 ΔA/h ml ≤ EPA ≤ 250 ΔA/h ml), middle (250 ΔA/h ml < EPA ≤ 500 ΔA/h ml) and high (EPA > 500 ΔA/h ml) peptidase producers.

## Results

### *Pseudomonas* Phylogenomy

Evolutionary relationships between the species of the genus *Pseudomonas* were estimated by a phylogenomic treeing approach. As of March 2019 the List of Prokaryotic names with Standing in Nomenclature (LPSN; Parte, 2014) contained 188 *Pseudomonas* species with validly described names, of which *P. brassicacearum*, *P. chlororaphis*, *P. oleovorans*, and *P. syringae* can be further divided into nine subspecies (Supplementary Table S5). A total of 185 type strains representing 87.8% (165) of the *Pseudomonas* species with validly published names were used and a maximum-likelihood (ML) phylogenomic tree was calculated on the basis of 92 universal bacterial core genes (Figure 1).

Based on the topology of the phylogenomy, 175 type strains were assigned to 21 monophyletic groups suggested previously by means of multi-locus-sequence-analysis (MLSA) (Mulet et al., 2010; Gomila et al., 2015; Peix et al., 2018). In addition, a new group (*P. alcaligenes*) consisting of the type strains *P. alcaligenes* NBRC 14159^T^, *P. fluvialis* ASS-1^T^ and *P. pharmafabricae* ZYSR67-Z^T^ was formed. With few exceptions, group memberships remained unchanged compared to the above mentioned MLSA analysis. Only two type strains changed their position and were moved from the *P. aeruginosa* monophylum to the *P. anguilliseptica* (*P. cuatrocienegasensis* CIP 109853^T^) and *P. alcaligenes* (*P. alcaligenes* NBRC 14159^T^) group. Furthermore, two type strains without former group affiliation (*P. aestusnigri* CECT 8317^T^ and *P. salegens* CECT 8338^T^) were placed in the *P. pertucinogena* group. In total, 23 of the type strains investigated were not part of previous MLSA phylogenetic studies. Supplementary Table S6 lists the phylogenomic group memberships of these strains.

### Distribution of the *aprX* Gene Within the Genus

To shed light on the distribution of the *aprX* gene within the genus, protein-coding sequences of the 185 type strains were screened for the peptidase AprX using BLASTp (Camacho et al., 2009). Homologs were found in 81 type strains (black triangles in Figure 1). In five of these strains, genome assemblies indicate that the *aprX* genes might not be functional due to the occurrence of mutations (Supplementary Table S7). Hence, the overall frequency of *aprX* carrying type strains in the genus corresponds to 43.8%. However, type strains harboring *aprX* are not evenly distributed across the genus. The presence of *aprX* is strongly associated to the monophylum composed of the phylogenetic groups *P. fluorescens*, *P. gessardii*, *P. fragi*, *P. mandelii*, *P. jessenii*, *P. koreensis*, *P. corrugata*, *P. chlororaphis*, *P. asplenii*, *P. syringae*, and *P. lutea* (Fisher’s exact test, *p* $<2.2\;x\;10^{-16}$, odds-ratio ~52.1). Indeed, 83% (73 of 88) of the monophylum’s type strains carry *aprX*, whereas the gene is only present within 8.2% (8 of 97) of the remaining type strains (Figure 1). Apart from few exceptions (*P. fragi* and *P. putida*), *aprX* is present (≥80%) or absent (≤20%) in almost all members of the different monophyletic groups (Supplementary Table S8).

For 17 type strains from species (stars in Figure 1), which were shown to be milk-relevant in our previous study (von Neubeck et al., 2015), two strong associations were inferable: first, type strains of milk-relevant species are significantly associated to the clade consisting of the monophyletic groups *P. fluorescens*, *P. gessardii*, and *P. fragi* (Fisher’s exact test, *p* $\sim 1.5\;x\;10^{-9}$, odds-ratio ~39.7). In fact, only two type strains (*P. aeruginosa* DSM 50071^T^ and *P. protegens* CHA0^T^) of species classified as milk-relevant are not members of that clade. Second, the presence of *aprX* is significantly associated to milk-relevance (Fisher’s exact test, *p* $\sim 7.9\;x\;10^{-6}$), odds-ratio ~25.0. Of all type strains from milk-relevant species, *Pseudomonas helleri* DSM 29165^T^ is the only one without the genetic information for the *aprX* locus.

### Diversity of *aprX-lipA2* Genetic Compositions

The 81 type strains possessing *aprX*, as well as 86 additional strains mainly originating from raw milk samples were BLAST screened for the presence of the nine possible *aprX-lipA2* operon genes (*aprX*, *aprI*, *aprD*, *aprE*, *aprF*, *prtA*, *prtB*, *lipA1*, and *lipA2*). In total, 22 different *aprX-lipA2* genetic organizations were found among the investigated strains (Supplementary Table S9), including cases in which particular genes or gene cluster are detached from the operon and translocated to other positions in the genome (Figure 2). With the exception of *P. saponiphila* DSM 9751^T^, all strains containing *aprX* are carrying the genetic information for *aprI*, *aprD*, *aprE*, and *aprF* as well. In more than 95% of cases, all five genes are lying in close proximity to each other. Therefore, these genes can be considered stable core components of the operon across species boundaries. In contrast, the remaining four genes (*prtA*, *prtB*, *lipA1*, and *lipA2*) are much more variable with respect to their presence and order in the operon and may even occur at different genomic locations or be completely absent. Mostly, strains belonging to the same species exhibit the same combination of *aprX-lipA2* genes in identical gene clusters. There are only a few cases, in which members of the species *P. lundensis*, *P. fragi*, and *Pseudomonas* sp. 12 have varying *aprX-lipA2* genetic compositions (*P. lundensis* WS 5095 and *Pseudomonas* sp. DSM 29142, *P. fragi* B25^T^, *P. fragi* WS 5087, *P. fragi* WS 5094, and *P. fragi* WS 5120). Figure 3 illustrates the four most abundant genetic organizations (type 1–4) observed in the genus, namely *aprXIDEF prtAB lipA2* (28 species), *aprXIDEF lipA2* (13 species), *aprXIDEF | prtAB* (11 species), and *aprXIDEF prtAB lipA1A2* (eight species). Together they account for more than two-thirds of all species that contain the operon or at least parts of it (Supplementary Table S9).

### Correlation Between Peptidase Activity and *aprX-lipA2* Variants

In order to study the relationship between *aprX-lipA2* operon structure and peptidase production, the proteolytic activity of 129 *Pseudomonas* strains possessing diverse operon types was screened after 3, 4, and 7 days of growth by using an agar diffusion assay. Besides 46 type strains, the strain set contained 83 additional isolates, mainly from raw milk (Supplementary Table S2).

**Figure 4:**
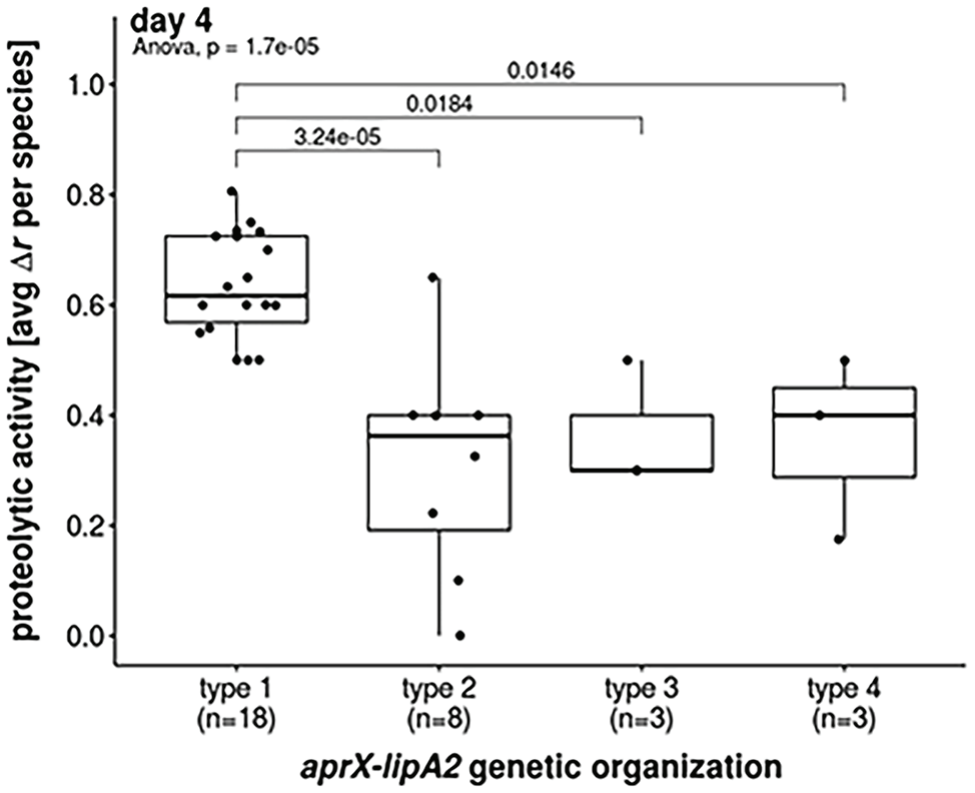
Proteolytic activities of most abundant *aprX-lipA2* genetic organizations. Boxplot representation of proteolytic activities at day 4 with regard to the most abundant *aprX-lipA2* genetic organizations, namely *aprXIDEF prtAB lipA2* (type 1), *aprXIDEF lipA2* (type 2), *aprXIDEF | prtAB* (type 3) and *aprXIDEF prtAB lipA1A2* (type 4). Each dot refers to the average proteolytic activity of a particular *Pseudomonas* species. One-way ANOVA revealed a significant difference in mean proteolytic activity between *aprX-lipA2* genetic organizations (*p* =1.7 x 10^-5^). *Post hoc* Tukey’s Test indicates that significant differences exist between type 1 and all other genetic organizations.

The strains split into 88 isolates exhibiting proteolysis on skim milk agar and 41 strains for which no clearing zone was observable. The activity of all proteolytic strains, except for *P. deceptionensis* DSM 26521^T^, increased over time, although this rise was strongly species‐ and partly strain-dependent (Figure 2). In general, the following trends for correlating the most common operon variants (type 1–4) with proteolytic activity became apparent. As expected, all 19 tested strains missing the *aprX-lipA2* genes were non-proteolytic. In contrast, 43 isolates harboring the operon of type 1 (*aprXIDEF prtAB lipA2*) revealed the strongest proteolytic activity in our study. Mean values were significantly higher than those of strains with genetic organizations of type 2, 3, and 4 (Figure 4).

Moreover, for operon type 1 candidates, a distinction between members of the *P. gessardii* and *P. fluorescens* group was observed: In case of the *P. gessardii* group, 74% of strains were already highly proteolytic on day 3 and 96% showed very high proteolytic activity on day 7. In the *P. fluorescens* group, 62% of the members were moderately proteolytic on day 3 and only 53% very highly proteolytic on day 7 (Figure 2). Additionally, five out of six *P. lactis* and *Pseudomonas* sp. 12 strains with operon type 9, which differs from structure type 1 only by an additional *lipA1* gene in the chromosome, were strongly proteolytic on day 7. Finally, also isolates of *P. protegens*, carrying *lipA1* upstream of *prtAB* (type 12), showed high or very high proteolytic activity.

In contrast to the highly proteolytic strains, isolates with the *prtAB*-lacking operon structure *aprXIDEF lipA2* (type 2), in general, had the lowest peptidase activities in our study. However, for the 23-type 2-member two major groups could be discriminated according to their phylogenetic distance and proteolytic behavior. While all 13 isolates of *P. fragi*, *P. deceptionensis*, *P. lundensis*, and *Pseudomonas* sp. 2, belonging to the *P. fragi* subgroup, displayed no to moderate proteolytic activity, the nine tested members of *P. veronii*, *P. grimontii*, *P. marginalis*, and *P. panacis*, which are part of the *P. fluorescens* subgroup, were mostly highly proteolytic at day 7.

Remarkably, the five tested isolates with the type 3 structure (*aprXIDEF | prtAB*), which have the genes *prtAB* located outside of the *aprX-lipA2* gene cluster, showed very diverse proteolytic potentials on skim milk agar, ranging from no to high enzyme activity at day 7, even for three very closely related strains of the *P. fragi* subgroup (Figure 2). However, for members of *P. lundensis* and *P. weihenstephanensis* with operon type 8, having also the *prtAB* genes located separately plus an additional *lipA2* gene downstream of *aprF*, the majority (9 out of 13 strains) did not show any proteolytic activity.

Finally, seven strains of the *P. chlororaphis*, *P. koreensis*, and *P. mandelii* subgroups, possessing the type 4 operon (*aprXIDEF prtAB lipA1A2*) with an additional lipase gene (*lipA1*) downstream of *prtAB*, revealed also highly strain-specific, but in general reduced proteolytic activities compared to isolates missing the extra lipase gene in the operon. Interestingly, several type strains (e.g., *P. aeruginosa* DSM 50071^T^, *P. fluorescens* DSM 50090^T^, *P. gessardii* DSM 17152^T^, *P. koreensis* DSM 16610^T^, *P. mandelii* DSM 17967^T^, and *P. poae* DSM 14936^T^), which did not originate from milk, exhibited considerably lower proteolytic activities than corresponding milk isolates, suggesting a possible influence of environmental adaptation on the proteolytic potential of single isolates.

### Quantitative Analysis of Proteolytic Activity in Milk Relevant *Pseudomonas* Species

To analyze AprX production under more realistic conditions, selected strains were cultivated in milk and peptidase activity was determined using the azocasein assay. In contrast to the screening assay on skim milk agar, this method allowed a more sensitive and specific quantification of AprX activity, since cells and supernatant were separated and thus only secreted enzymes were measured. Although bacteria were cultivated at 12°C for both assays, the azocasein assay quantified the substrate turnover of extracellular proteases after incubation of 1 h at 40°C, which corresponds more closely to the temperature optimum of AprX and the storage conditions of UHT milk products in case of long transport routes. In total, 28 strains (assigned to 18 different *Pseudomonas* species) with various operon types were selected, incubated for 4 days in UHT milk and the peptidase activity was determined after day 3 and 4.

**Figure 5:**
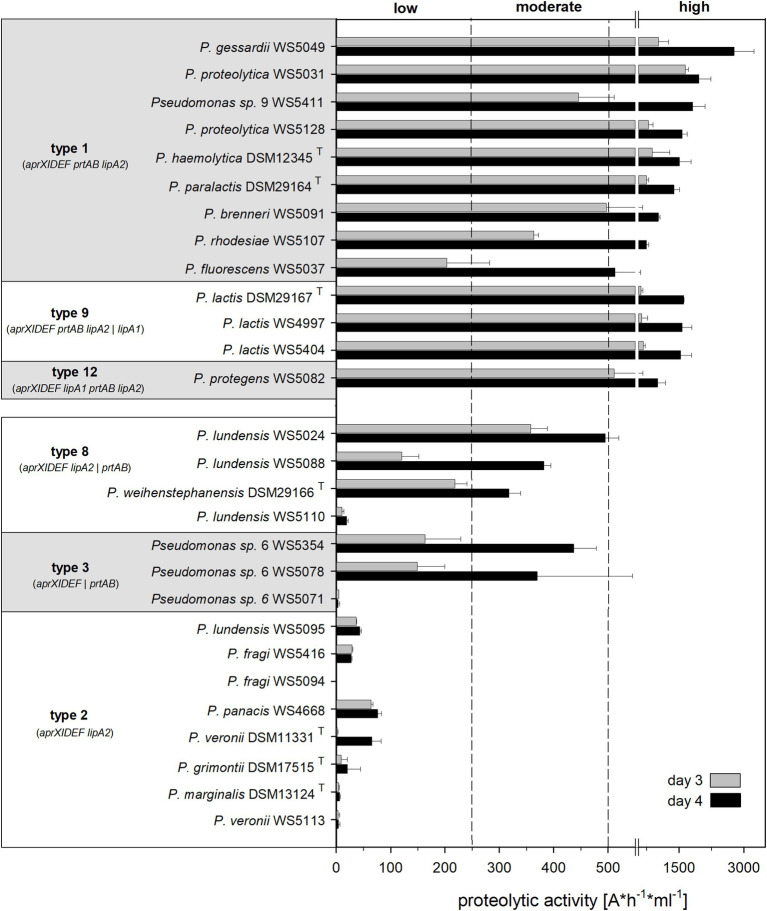
Proteolytic activity of 28 *Pseudomonas* strains, grouped according to their *aprX-lipA2* genetic organization into highly proteolytic (type 1, 9, 12) and middle to low proteolytic isolates (type 2, 3, 8). Extracellular peptidase activity of strains, grown in UHT milk at 12°C, was determined after day 3 and 4 *via* azocasein assay. Each strain was cultivated in triplicates and EPA measurement was carried out in duplicates for each sample; means were calculated and standard deviations are shown as error bars. Dashed lines point out the classification into low, moderate and highly proteolytic isolates according to proteolytic activity values determined on day 4.

While the proteolytic activity enhanced from day 3 to 4 between 1.7‐ and 4-fold for most of the strains tested, the degree of increase was strongly strain-dependent (Figure 5). All isolates harboring genetic organizations of type 1, 9, or 12 were strongly proteolytic and thus defined to be “high producers” (EPA > 500 Δ A/h ml), largely confirming the results based on the agar diffusion assay (Figures 4, 5). Among them, *P. gessardii* WS 5049 exhibited the highest measured proteolytic activity on day 4 (2,775 ΔA x h^−1^ x ml^−1^).

Similarly, the eight isolates with an operon structure of type 2 (*aprXIDEF lipA2*) were classified as “low producers” in the azocasein assay (Figure 5). Here, all type 2 members, though belonging to phylogenetically different subgroups (*P. fragi* and *P. fluorescens*), showed very similar proteolytic behavior, which was not observed so clearly in the agar diffusion assay. Moreover, in the azocasein assay, five out of seven tested strains, which have the *prtAB* genes located outside of the operon (type 3 and 8), turned out to be “moderate producers,” indicating a potential role of PrtAB on peptidase activity (Figure 5). These findings partly differ from the results of the agar diffusion test, where most of these strains were non-proteolytic, possibly due to other assay conditions or the lower sensitivity of this method.

## Discussion

In the genus *Pseudomonas*, the expression of heat-resistant enzymes, especially the AprX peptidase, is decisive for the milk spoilage potential of strains. In this study, we assigned the presence of the *aprX*-gene to a distinct monophyletic clade comprising 88 species within the genus *Pseudomonas*. Milk relevant species were mainly allocated to the *P. gessardii*, *P. fluorescens*, and *P. fragi* subgroups. In total, 22 different *aprX-lipA2* genetic organizations were identified, substantially expanding previous work on *aprX-lipA2* operon structures in various *Pseudomonas* species. A study by Ma et al. described five different *aprX-lipA2* operon variants in six strains, which share, analogous to our findings, a set of core genes (*aprXIDEF*) and up to four additional genes (*lipA1*, *lipA2*, *prtA*, and *prtB*) (Ma et al., 2003). Our results are in good agreement, although we found only one operon type for *P. fluorescens* strains, but in total five for the whole *P. fluorescens* subgroup, while former studies revealed three different structures for the species *P. fluorescens* (Woods et al., 2001; Ma et al., 2003). These differences are likely due to difficulties in assigning *Pseudomonas* isolates to the accurate species, especially within subgroups of very closely related members.

Analysis of the proteolytic activity using agar diffusion and azocasein assay showed significant and strain-specific differences among the isolates tested, but several general correlations with the various *aprX-lipA2* genetic compositions were found. In both assays, members of the *P. gessardii* and *P. fluorescens* subgroup with a type 1 (*aprXIDEF prtAB lipA2*) or a type 9 (*aprXIDEF prtAB lipA2 | lipA1*) operon structure reached highest proteolytic activities, while isolates containing alternative *aprX-lipA2* genetic organizations and mostly belonging to other subgroups, e.g., *P. fragi*, were much less proteolytic. This is in line with previous studies showing that proteolytic strains were frequently assigned to species of the *P. gessardii* or *P. fluorescens* subgroup and that they exhibited significantly higher enzyme activities than isolates of the *P. fragi* subgroup (Wiedmann et al., 2000; von Neubeck et al., 2015; Glück et al., 2016).

Furthermore, our study revealed that especially members of the *P. fragi* subgroup like *P. lundensis* and phylogenetically proximate species are variable in their genetic organization of the *aprX-lipA2* operon and proteolytic activity was for the most part strain-dependent. However, *via* azocasein assay, it was seen that strains of this subgroup, which possessed the *prtAB* genes apart from the *aprX-lipA2* operon (type 3, *aprXIDEF | prtAB* and type 8, *aprXIDEF lipA2 | prtAB*), revealed higher proteolytic activity compared to strains lacking these genes completely (type 2, *aprXIDEF lipA2*). Therefore, and since strains with the operon structure of type 1, containing *prtAB* within the operon, were mostly highly proteolytic, a potential influence of *prtA* and *prtB* on the proteolytic activity can be suspected. The proteins PrtA (PspA) and PrtB (PspB) are described as putative autotransporters partly homologs to a serine protease found in *Serratia marcescens* (Kawai et al., 1999; Ma et al., 2003; Sun and Sun, 2015). So far, it was not shown that PrtA or PrtB themselves have peptidase activity in *Pseudomonas fluorescens*, but it was suggested that these proteins could be involved as autoaggregation factors in adhesion and are important for biofilm formation (Chabeaud et al., 2001; Woods et al., 2001). A link between the production of biofilm and extracellular enzymes has been shown before for several bacteria like *Aeromonas hydrophila*, *Vibrio parahaemolyticus*, or *Pseudomonas* (Khajanchi et al., 2009; Teh et al., 2012; Mizan et al., 2016). However, since we measured the proteolytic activity of planktonic cells in the azocasein assay, there might be another role of PrtA and PrtB regarding AprX synthesis, independent of biofilm formation. Therefore, our further work will focus on elucidating the function of these proteins on peptidase production of *Pseudomonas* in more detail.

Besides various structures of the *aprX-lipA2* operon and a potential role of PrtAB, diverse enzyme properties of AprX itself may be another reason for variations of proteolytic activity among species assigned to different subgroups. In previous studies, a discrimination between AprX peptidases of group 1 (from *P. lundensis* and *P. weihenstephanensis* strains) and AprX of group 2 (from *P. proteolytica*, *P. lactis*, and *P. paralactis* strains) was conducted. Although sharing an amino acid sequence similarity of 68%, group 2 enzymes exhibited a 3–30-fold activity compared to peptidases of group 1 under optimal conditions for the respective enzyme (Glück et al., 2016; Volk et al., 2019). These findings support our data, revealing significant higher proteolytic activities for members of the *P. gessardii* and *P. fluorescens* subgroup (encompassing *P. proteolytica*, *P. lactis*, and *P. paralactis*) than for those of the *P. fragi* subgroup including *P. lundensis* and *P. weihenstephanensis*. Varying substrate turnover rates of AprX may also contribute to the observed higher peptidase activity from several members of the *P. fluorescens* subgroup (e.g., *P. veronii*, *P. grimontii*, *P. panacis*, and *P. marginalis*) in comparison to isolates of the *P. fragi* subgroup, though having the same operon structure of type 2. However, this effect was only observed in the agar diffusion assay, whereas in the azocasein assay both groups were not clearly distinguishable with respect to their proteolytic activity, suggesting an interplay of many factors influencing protease activity. In order to investigate further the degree of milk spoilage caused by different AprX proteins, more complex enzyme tests, using milk as substrate and detecting the release of α-amino groups for quantification of casein degradation would be necessary.

In general, inconsistent results from the two methods we used for detecting proteolytic activity occurred mainly for the non‐ to middle proteolytic isolates and may be due to diverse detection limits and different growth conditions, especially temperatures applied in both assays. In the literature, several environmental factors were demonstrated to affect the production or activity of AprX in *Pseudomonas* strains, including temperature (Burger et al., 2000; Nicodeme et al., 2005; Meng et al., 2018), medium composition (Martins et al., 2014), iron content (Woods et al., 2001; Maunsell et al., 2006), and calcium concentration (Ertan et al., 2015; for review see Zhang et al., 2019). Recent studies stated that temperature and pH-value have a major influence on the substrate turnover rates of AprX and showed that the optimal temperature‐ and pH-values vary greatly for distinct peptidases isolated from different *Pseudomonas* species, even partly independent from their phylogenetic position (Baur et al., 2015b; Glück et al., 2016; Volk et al., 2019). In addition, several works identified *aprX* as a target gene of the global GacS/GacA two-component system, a main regulator of secondary metabolism in *Pseudomonas* (Blumer et al., 1999; Heeb et al., 2002; Hassan et al., 2010), emphasizing once more a multifactorial network controlling peptidase production.

In summary, our study demonstrates a large genetic variety of *aprX-lipA2* genetic organizations in the genus *Pseudomonas* and indicates a correlation between the proteolytic activity, the *aprX-lipA2* operon structure and the phylogenetic position of the producer. Besides such intrinsic factors, several external conditions seem to influence synthesis and activity of AprX causing strain-specific proteolytic profiles. The underlying regulatory mechanisms appear to be rather complex and need to be elucidated in future studies for a better understanding and prediction of peptidase production in *Pseudomonas*, especially for milk-relevant *Pseudomonas* species.

## Data Availability Statement

The original contributions presented in the study are publicly available. This data can be found in NCBI under PRJNA612806.

## Author Contributions

CM: library preparation, genome sequencing, agar diffusion assays, azocasein assays, data analysis and visualization, manuscript drafting, and writing. CH: library preparation, genome sequencing, pyhlogenomic analysis, bioinformatics analysis, statistical analysis, data analysis and visualization, manuscript drafting, and writing. MN: library preparation, genome sequencing, and data analysis. SS: data discussion, manuscript revision. MW: data discussion, manuscript revision, project planning. GL: data discussion, manuscript revision, project supervision. All authors read and approved the final manuscript.

## Conflict of Interest

MN is currently employed by the company Eurofins BioPharma Product Testing Munich GmbH.The remaining authors declare that the research was conducted in the absence of any commercial or financial relationships that could be construed as a potential conflict of interest.

## Acknowledgments

We would like to thank Sonja Dandl, Charon Fuhrmann and Inge Celler for excellent technical support. This IGF Project of the FEI was supported *via* AiF within the programme for promoting the Industrial Collective Research (IGF) of the German Ministry of Economic Affairs and Energy (BMWi), based on a resolution of the German Parliament.

## Supplementary Material

The Supplementary Material for this article can be found online at: https://www.frontiersin.org/articles/10.3389/fmicb.2020.01190/full#supplementary-material.

## Footnotes

^1^https://www.bioinformatics.babraham.ac.uk/projects/fastqc/

## References

[ref1] Alves M. P. Salgado R. L. Eller M. R. Dias R. S. Oliveira de Paula S. Fernandes de Carvalho A. (2018). Temperature modulates the production and activity of a metalloprotease from *Pseudomonas fluorescens* 07A in milk. J. Dairy Sci. 101, 992–999. 10.3168/jds.2017-13238, PMID: 29248219

[ref2] Arnold J. B. (2019). *ggthemes: extra themes, scales and geoms for ‘ggplot2’* [Online]. Available at: https://CRAN.R-project.org/package=ggthemes (Accessed March 19, 2020).

[ref3] Auguie B. (2017). *gridExtra: miscellaneous functions for "Grid" graphics* [Online]. Available at: https://CRAN.R-project.org/package=gridExtra (Accessed March 19, 2020).

[ref4] Bankevich A. Nurk S. Antipov D. Gurevich A. A. Dvorkin M. Kulikov A. S.. (2012). SPAdes: a new genome assembly algorithm and its applications to single-cell sequencing. J. Comput. Biol. 19, 455–477. 10.1089/cmb.2012.0021, PMID: 22506599 PMC3342519

[ref6] Baur C. Krewinkel M. Kutzli I. Kranz B. von Neubeck M. Huptas C. (2015b). Isolation and characterisation of a heat-resistant peptidase from *Pseudomonas panacis* withstanding general UHT processes. Int. Dairy J. 49, 46–55. 10.1016/j.idairyj.2015.04.009

[ref5] Baur C. Krewinkel M. Kranz B. von Neubeck M. Wenning M. Scherer S. (2015a). Quantification of the proteolytic and lipolytic activity of microorganisms isolated from raw milk. Int. Dairy J. 49, 23–29. 10.1016/j.idairyj.2015.04.005

[ref7] Blumer C. Heeb S. Pessi G. Haas D. (1999). Global GacA-steered control of cyanide and exoprotease production in *Pseudomonas fluorescens* involves specific ribosome binding sites. PNAS 96, 14073–14078. 10.1073/pnas.96.24.14073 10570200 PMC24192

[ref8] Burger M. Woods R. G. McCarthy C. Beacham I. R. (2000). Temperature regulation of protease in *Pseudomonas fluorescens* LS107d2 by an ECF sigma factor and a transmembrane activator. Microbiology 146, 3149–3155. 10.1099/00221287-146-12-3149, PMID: 11101673

[ref9] Camacho C. Coulouris G. Avagyan V. Ma N. Papadopoulos J. Bealer K.. (2009). BLAST+: architecture and applications. BMC Bioinformatics 10:421. 10.1186/1471-2105-10-421, PMID: 20003500 PMC2803857

[ref10] Chabeaud P. de Groot A. Bitter W. Tommassen J. Heulin T. Achouak W. (2001). Phase-variable expression of an operon encoding extracellular alkaline protease, a serine protease homolog, and lipase in *Pseudomonas brassicacearum*. J. Bacteriol. 183, 2117–2120. 10.1128/JB.183.6.2117-2120.2001, PMID: 11222613 PMC95110

[ref11] Dufour D. Nicodeme M. Perrin C. Driou A. Brusseaux E. Humbert G.. (2008). Molecular typing of industrial strains of *Pseudomonas* spp. isolated from milk and genetical and biochemical characterization of an extracellular protease produced by one of them. Int. J. Food Microbiol. 125, 188–196. 10.1016/j.ijfoodmicro.2008.04.004, PMID: 18511140

[ref12] Duong F. Bonnet E. Geli V. Lazdunski A. Murgier M. Filloux A. (2001). The AprX protein of *Pseudomonas aeruginosa*: a new substrate for the Apr type I secretion system. Gene 262, 147–153. 10.1016/S0378-1119(00)00541-2, PMID: 11179678

[ref13] Ertan H. Cassel C. Verma A. Poljak A. Charlton T. Aldrich-Wright J. (2015). A new broad specificity alkaline metalloprotease from a *Pseudomonas* sp. isolated from refrigerated milk: role of calcium in improving enzyme productivity. J. Mol. Catal. B Enzym. 113, 1–8. 10.1016/j.molcatb.2014.12.010

[ref14] Fox J. Weisberg S. (2019). An R companion to applied regression. Thousand Oaks, CA: Sage.

[ref15] Glück C. Rentschler E. Krewinkel M. Merz M. von Neubeck M. Wenning M. (2016). Thermostability of peptidases secreted by microorganisms associated with raw milk. Int. Dairy J. 56, 186–197. 10.1016/j.idairyj.2016.01.025

[ref16] Gomila M. Pena A. Mulet M. Lalucat J. Garcia-Valdes E. (2015). Phylogenomics and systematics in *Pseudomonas*. Front. Microbiol. 6:214. 10.3389/fmicb.2015.00214, PMID: 26074881 PMC4447124

[ref17] Guzzo J. Duong F. Wandersman C. Murgier M. Lazdunski A. (1991). The secretion genes of *Pseudomonas aeruginosa* alkaline protease are functionally related to those of Erwinia chrysanthemi proteases and *Escherichia coli* alpha-haemolysin. Mol. Microbiol. 5, 447–453. 10.1111/j.1365-2958.1991.tb02128.x, PMID: 1904127

[ref18] Hassan K. A. Johnson A. Shaffer B. T. Ren Q. Kidarsa T. A. Elbourne L. D.. (2010). Inactivation of the GacA response regulator in *Pseudomonas fluorescens* Pf-5 has far-reaching transcriptomic consequences. Environ. Microbiol. 12, 899–915. 10.1111/j.1462-2920.2009.02134.x, PMID: 20089046

[ref19] Heeb S. Blumer C. Haas D. (2002). Regulatory RNA as mediator in GacA/RsmA-dependent global control of exoproduct formation in *Pseudomonas fluorescens* CHA0. J. Bacteriol. 184, 1046–1056. 10.1128/jb.184.4.1046-1056.2002, PMID: 11807065 PMC134805

[ref20] Huptas C. Scherer S. Wenning M. (2016). Optimized Illumina PCR-free library preparation for bacterial whole genome sequencing and analysis of factors influencing de novo assembly. BMC Res. Notes 9:269. 10.1186/s13104-016-2072-9 27176120 PMC4864918

[ref21] Hyatt D. Chen G. L. Locascio P. F. Land M. L. Larimer F. W. Hauser L. J. (2010). Prodigal: prokaryotic gene recognition and translation initiation site identification. BMC Bioinformatics 11:119. 10.1186/1471-2105-11-119 20211023 PMC2848648

[ref22] Kassambara A. (2019a). *ggpubr: ‘ggplot2’ based publication ready plots* [Online]. Available at: https://CRAN.R-project.org/package=ggpubr (Accessed March 19, 2020).

[ref23] Kassambara A. (2019b). *rstatix: pipe-friendly framework for basic statistical tests* [Online]. Available at: https://CRAN.R-project.org/package=rstatix (Accessed March 19, 2020).

[ref24] Kawai E. Idei A. Kumura H. Shimazaki K.-i. Akatsuka H. Omori K. (1999). The ABC-exporter genes involved in the lipase secretion are clustered with the genes for lipase, alkaline protease, and serine protease homologues in *Pseudomonas fluorescens* no. 33. Biochim. Biophys. Acta 1446, 377–382. 10.1016/S0167-4781(99)00094-9, PMID: 10524213

[ref25] Khajanchi B. K. Sha J. Kozlova E. V. Erova T. E. Suarez G. Sierra J. C.. (2009). N-acylhomoserine lactones involved in quorum sensing control the type VI secretion system, biofilm formation, protease production, and in vivo virulence in a clinical isolate of *Aeromonas hydrophila*. Microbiology 155, 3518–3531. 10.1099/mic.0.031575-0, PMID: 19729404 PMC2888131

[ref26] Kumar S. Stecher G. Li M. Knyaz C. Tamura K. (2018). MEGA X: molecular evolutionary genetics analysis across computing platforms. Mol. Biol. Evol. 35, 1547–1549. 10.1093/molbev/msy096, PMID: 29722887 PMC5967553

[ref27] Letunic I. Bork P. (2019). Interactive tree of life (iTOL) v4: recent updates and new developments. Nucleic Acids Res. 47, W256–W259. 10.1093/nar/gkz239, PMID: 30931475 PMC6602468

[ref28] Liao C. H. McCallus D. E. (1998). Biochemical and genetic characterization of an extracellular protease from *Pseudomonas fluorescens* CY091. Appl. Environ. Microbiol. 64, 914–921. 10.1128/AEM.64.3.914-921.1998, PMID: 9501431 PMC106346

[ref29] Liu M. Wang H. Griffiths M. W. (2007). Regulation of alkaline metalloprotease promoter by N-acyl homoserine lactone quorum sensing in *Pseudomonas fluorescens*. J. Appl. Microbiol. 103, 2174–2184. 10.1111/j.1365-2672.2007.03488.x, PMID: 18045400

[ref30] Ma Q. Zhai Y. Schneider J. C. Ramseier T. M. Saier M. H. Jr. (2003). Protein secretion systems of *Pseudomonas aeruginosa* and *P. fluorescens*. Biochim. Biophys. Acta 1611, 223–233. 10.1016/s0005-2736(03)00059-2 12659964

[ref31] Marchand S. Vandriesche G. Coorevits A. Coudijzer K. De Jonghe V. Dewettinck K.. (2009). Heterogeneity of heat-resistant proteases from milk *Pseudomonas* species. Int. J. Food Microbiol. 133, 68–77. 10.1016/j.ijfoodmicro.2009.04.027, PMID: 19481283

[ref32] Martins M. L. Pinto U. M. Riedel K. Vanetti M. C. (2015). Milk-deteriorating exoenzymes from *Pseudomonas fluorescens* 041 isolated from refrigerated raw milk. Braz. J. Microbiol. 46, 207–217. 10.1590/S1517-838246120130859, PMID: 26221110 PMC4512081

[ref33] Martins M. L. Pinto U. M. Riedel K. Vanetti M. C. D. Mantovani H. C. de Araújo E. F. (2014). Lack of AHL-based quorum sensing in *Pseudomonas fluorescens* isolated from milk. Braz. J. Microbiol. 54, 1039–1046. 10.1590/s1517-83822014000300037 PMC4204945 25477941

[ref34] Matéos A. Guyard-Nicodème M. Baglinière F. Jardin J. Gaucheron F. Dary A. (2015). Proteolysis of milk proteins by AprX, an extracellular protease identified in *Pseudomonas* LBSA1 isolated from bulk raw milk, and implications for the stability of UHT milk. Int. Dairy J. 49, 78–88. 10.1016/j.idairyj.2015.04.008

[ref35] Maunsell B. Adams C. O’Gara F. (2006). Complex regulation of AprA metalloprotease in *Pseudomonas fluorescens* M114: evidence for the involvement of iron, the ECF sigma factor, PbrA and pseudobactin M114 siderophore. Microbiology 152, 29–42. 10.1099/mic.0.28379-0, PMID: 16385113

[ref36] McKellar R. C. (1981). Development of off-flavors in ultra-high temperature and pasteurized milk as a function of proteolysis. J. Dairy Sci. 64, 2138–2145. 10.3168/jds.S0022-0302(81)82820-2

[ref37] Meng L. Liu H. Dong L. Zheng N. Xing M. Zhang Y.. (2018). Identification and proteolytic activity quantification of *Pseudomonas* spp. isolated from different raw milks at storage temperatures. J. Dairy Sci. 101, 2897–2905. 10.3168/jds.2017-13617, PMID: 29398021

[ref38] Mizan M. F. Jahid I. K. Kim M. Lee K. H. Kim T. J. Ha S. D. (2016). Variability in biofilm formation correlates with hydrophobicity and quorum sensing among *Vibrio parahaemolyticus* isolates from food contact surfaces and the distribution of the genes involved in biofilm formation. Biofouling 32, 497–509. 10.1080/08927014.2016.1149571, PMID: 26980068

[ref39] Mulet M. Lalucat J. Garcia-Valdes E. (2010). DNA sequence-based analysis of the *Pseudomonas* species. Environ. Microbiol. 12, 1513–1530. 10.1111/j.1462-2920.2010.02181.x, PMID: 20192968

[ref40] Na S. I. Kim Y. O. Yoon S. H. Ha S. M. Baek I. Chun J. (2018). UBCG: up-to-date bacterial core gene set and pipeline for phylogenomic tree reconstruction. J. Microbiol. 56, 280–285. 10.1007/s12275-018-8014-6, PMID: 29492869

[ref41] von Neubeck M. Baur C. Krewinkel M. Stoeckel M. Kranz B. Stressler T.. (2015). Biodiversity of refrigerated raw milk microbiota and their enzymatic spoilage potential. Int. J. Food Microbiol. 211, 57–65. 10.1016/j.ijfoodmicro.2015.07.001, PMID: 26173200

[ref42] Nicodeme M. Grill J. P. Humbert G. Gaillard J. L. (2005). Extracellular protease activity of different *Pseudomonas* strains: dependence of proteolytic activity on culture conditions. J. Appl. Microbiol. 99, 641–648. 10.1111/j.1365-2672.2005.02634.x, PMID: 16108806

[ref43] Nikolenko S. I. Korobeynikov A. I. Alekseyev M. A. (2013). BayesHammer: Bayesian clustering for error correction in single-cell sequencing. BMC Genomics 14(Suppl 1):S7. 10.1186/1471-2164-14-S1-S7, PMID: 23368723 PMC3549815

[ref44] Palleroni N. J. (2015). “Genus I. *Pseudomonas Migula* 1894” in Bergey’s manual of systematic bacteriology, the proteobacteria, part B, the gammaproteobacteria. eds. Brenner D. J. Krieg N. R. Staley J. T., vol. 2 2nd Edn (New York, NY: Springer), 323–379.

[ref45] Parte A. C. (2014). LPSN—list of prokaryotic names with standing in nomenclature. Nucleic Acids Res. 42, D613–D616. 10.1093/nar/gkt1111 24243842 PMC3965054

[ref46] Patel R. K. Jain M. (2012). NGS QC toolkit: a toolkit for quality control of next generation sequencing data. PLoS One 7:e30619. 10.1371/journal.pone.0030619, PMID: 22312429 PMC3270013

[ref47] Peix A. Ramirez-Bahena M. H. Velazquez E. (2018). The current status on the taxonomy of *Pseudomonas* revisited: an update. Infect. Genet. Evol. 57, 106–116. 10.1016/j.meegid.2017.10.026, PMID: 29104095

[ref48] R Core Team (2019). *R: a language and environment for statistical computing* [Online]. Available at: https://www.R-project.org/ (Accessed March 19, 2020).

[ref49] Schokker E. P. van Boekel M. A. J. S. (1997). Production, purification and partial characterization of the extracellular proteinase from *Pseudomonas fluorescens* 22F. Int. Dairy J. 7, 265–271. 10.1016/S0958-6946(97)00008-3

[ref50] Sievers F. Higgins D. G. (2014). Clustal omega, accurate alignment of very large numbers of sequences. Methods Mol. Biol. 1079, 105–116. 10.1007/978-1-62703-646-7_6 24170397

[ref51] Sørhaug T. Stepaniak L. (1997). Psychrotrophs and their enzymes in milk and dairy products: quality aspects. Trends Food Sci. Technol. 8, 35–41. 10.1016/S0924-2244(97)01006-6

[ref52] Stoeckel M. Lidolt M. Achberger V. Glück C. Krewinkel M. Stressler T. (2016). Growth of *Pseudomonas weihenstephanensis*, *Pseudomonas proteolytica* and *Pseudomonas* sp. in raw milk: impact of residual heat-stable enzyme activity on stability of UHT milk during shelf-life. Int. Dairy J. 59, 20–28. 10.1016/j.idairyj.2016.02.045

[ref53] Sun Y. Y. Sun L. (2015). *Pseudomonas fluorescens*: iron-responsive proteins and their involvement in host infection. Vet. Microbiol. 176, 309–320. 10.1016/j.vetmic.2015.01.020, PMID: 25680811

[ref54] Teh K. H. Flint S. Palmer J. Andrewes P. Bremer P. Lindsay D. (2012). Proteolysis produced within biofilms of bacterial isolates from raw milk tankers. Int. J. Food Microbiol. 157, 28–34. 10.1016/j.ijfoodmicro.2012.04.008, PMID: 22571990

[ref55] Varghese N. J. Mukherjee S. Ivanova N. Konstantinidis K. T. Mavrommatis K. Kyrpides N. C.. (2015). Microbial species delineation using whole genome sequences. Nucleic Acids Res. 43, 6761–6771. 10.1093/nar/gkv657, PMID: 26150420 PMC4538840

[ref56] Volk V. Gluck C. Leptihn S. Ewert J. Stressler T. Fischer L. (2019). Two heat resistant endopeptidases from *Pseudomonas* species with destabilizing potential during milk storage. J. Agric. Food Chem. 67, 905–915. 10.1021/acs.jafc.8b04802 30585481

[ref57] Wickham H. (2016). ggplot2: elegant graphics for data analysis. New York: Springer-Verlag.

[ref58] Wiedmann M. Weilmeier D. Dineen S. S. Ralyea R. Boor K. J. (2000). Molecular and phenotypic characterization of *Pseudomonas* spp. isolated from milk. Appl. Environ. Microbiol. 66, 2085–2095. 10.1128/AEM.66.5.2085-2095.2000, PMID: 10788386 PMC101459

[ref59] Wilke C. O. (2019). *cowplot: streamlined plot theme and plot annotations for ‘ggplot2’* [Online]. Available at: https://CRAN.R-project.org/package=cowplot (Accessed March 19, 2020).

[ref60] Woods R. G. Burger M. Beven C. A. Beacham I. R. (2001). The *aprX-lipA* operon of *Pseudomonas fluorescens* B52: a molecular analysis of metalloprotease and lipase production. Microbiology 147, 345–354. 10.1099/00221287-147-2-345, PMID: 11158351

[ref61] Zhang C. Bijl E. Svensson B. Hettinga K. (2019). The extracellular protease AprX from *Pseudomonas* and its spoilage potential for UHT Milk: a review. Compr. Rev. Food Sci. F. 18, 834–852. 10.1111/1541-4337.12452 33336988

