# Supplementary material for: Genetic Organization of the aprX-lipA2 Operon Affects the Proteolytic Potential of Pseudomonas Species in Milk
Source: Front Microbiol. 2020 Jun 10;11:1190. doi: 10.3389/fmicb.2020.01190 (PMC7298200; doi:10.3389/fmicb.2020.01190)
Supplement: Supplementary file 1 [file Data_Sheet_1.docx]

Supplementary Material

# Supplementary Figures and Tables

## Supplementary Figures


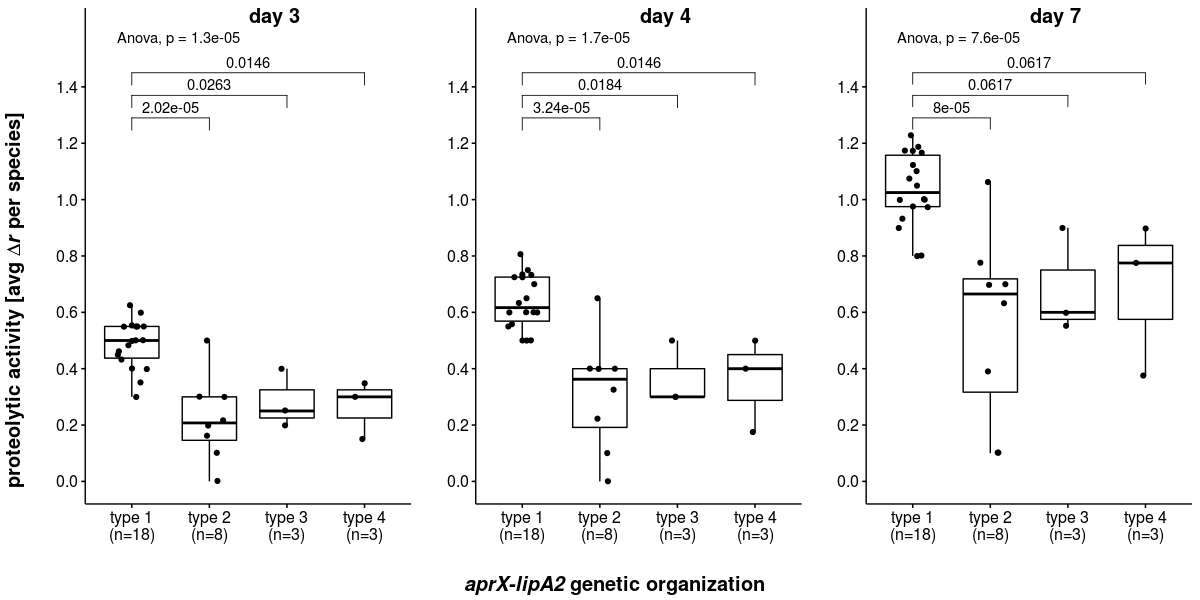


**Supplementary Figure 1.** Species-specific proteolytic activities at days 3, 4 and 7 plotted in dependence on the most abundant *aprX-lipA2* genetic organizations, namely *aprXIDEF prtAB lipA2* (type 1), *aprXIDEF lipA2* (type 2), *aprXIDEF | prtAB* (type 3) and *aprXIDEF prtAB lipA1A2* (type 4). One-way ANOVA statistical testing revealed a significant difference in mean proteolytic activity at each day. P-values resulting from post hoc tests (Tukey HSD) are shown for *aprXIDEF prtAB lipA2* (type 1) and the remaining genetic organizations.

## Supplementary Tables

**Supplementary Table 1.** *De novo* sequenced and assembled strains

| **Strain** | **sequenced**  **read length [bp]** | **high-quality**  **read length [bp]** | **sequencing**  **depth [x-fold]** | **N50** | **number**  **contigs** | **assembly**  **size** | **NCBI**  **WGS ID** |
| --- | --- | --- | --- | --- | --- | --- | --- |
| *P. aeruginosa* WS 5022 | 2x251 & 2x251 | 2x240 & 2x190 | 142 | 345,583 | 68 | 6,875,522 | JAAQZH01 |
| *P. brenneri* WS 5091 | 2x251 | 2x240 | 100 | 490,121 | 36 | 6,334,542 | JAAQZG01 |
| *P. chengduensis* DSM 26382 ^T^ | 2x251 | 2x240 | 116 | 267,193 | 59 | 5,445,831 | JAAQZF01 |
| *P. chlororaphis* WS 5014 | 2x251 | 2x240 | 39 | 1,036,757 | 31 | 6,787,621 | JAAQZE01 |
| *P. fluorescens* WS 5037 | 2x251 | 2x150 | 47 | 392,749 | 48 | 6,247,579 | JAAQZD01 |
| *P. fragi* WS 5025 | 2x251 & 2x251 | 2x240 & 2x190 | 210 | 559,105 | 12 | 4,993,846 | JAAQZC01 |
| *P. fragi* WS 5032 | 2x251 & 2x251 | 2x190 & 2x190 | 76 | 401,936 | 26 | 4,930,841 | JAAQZB01 |
| *P. fragi* WS 5045 | 2x251 & 2x251 | 2x190 & 2x190 | 81 | 573,481 | 17 | 5,025,573 | JAAQZA01 |
| *P. fragi* WS 5065 | 2x251 & 2x251 | 2x240 & 2x190 | 223 | 585,265 | 18 | 4,907,068 | JAAQYZ01 |
| *P. fragi* WS 5087 | 2x251 & 2x251 | 2x190 & 2x190 | 76 | 153,202 | 84 | 5,387,582 | JAAQYY01 |
| *P. fragi* WS 5094 | 2x251 & 2x251 | 2x190 & 2x190 | 62 | 123,552 | 97 | 5,333,538 | JAAQYX01 |
| *P. fragi* WS 5102 | 2x251 & 2x251 | 2x190 & 2x190 | 115 | 428,283 | 38 | 5,496,051 | JAAQYW01 |
| *P. fragi* WS 5112 | 2x251 & 2x251 | 2x190 & 2x190 | 69 | 378,318 | 55 | 5,456,846 | JAAQYV01 |
| *P. fragi* WS 5120 | 2x251 | 2x240 | 107 | 169,642 | 96 | 5,520,514 | JAAQYU01 |
| *P. fragi* WS 5123 | 2x251 | 2x240 | 101 | 761,139 | 13 | 4,814,352 | JAAQYT01 |
| *P. fragi* WS 5124 | 2x251 | 2x240 | 191 | 559,756 | 18 | 5,127,606 | JAAQYS01 |
| *P. fragi* WS 5125 | 2x251 | 2x240 | 57 | 823,015 | 27 | 4,973,746 | JAAQYR01 |
| *P. fragi* WS 5416 | 2x226 | 2x215 | 53 | 2,817,414 | 12 | 4,997,423 | JAAQYQ01 |
| *P. gessardii* WS 5409 | 2x251 | 2x240 | 136 | 90,666 | 183 | 6,933,365 | JAAQYP01 |
| *P. gessardii* WS 5049 | 2x251 | 2x240 | 100 | 169,792 | 89 | 6,737,829 | JAAQYO01 |
| *P. gessardii* WS 5408 | 2x226 | 2x215 | 26 | 112,571 | 125 | 6,660,153 | JAAQYN01 |
| *P. koreensis* DSM 16610 ^T^ | 2x251 | 2x240 | 100 | 689,935 | 27 | 6,086,647 | JAAQYM01 |
| *P. koreensis* WS 5015 | 2x251 | 2x240 | 42 | 892,370 | 17 | 6,035,452 | JAAQYL01 |
| *P. lactis* WS 4997 | 2x251 | 2x240 | 58 | 584,278 | 29 | 6,670,265 | JAAQYK01 |
| *P. lactis* WS 5000 | 2x226 | 2x215 | 62 | 892,675 | 26 | 6,536,092 | JAAQYJ01 |
| *P. lactis* WS 5404 | 2x275 | 2x225 | 80 | 392,731 | 46 | 6,852,845 | JAAQYI01 |
| *P. lactis* WS 5405 | 2x275 | 2x225 | 76 | 404,422 | 55 | 6,829,825 | JAAQYH01 |
| *P. lundensis* WS 5024 | 2x251 | 2x150 | 84 | 158,609 | 102 | 5,219,803 | JAAQYG01 |
| *P. lundensis* WS 5026 | 2x251 | 2x240 | 99 | 220,333 | 80 | 5,220,964 | JAAQYF01 |
| *P. lundensis* WS 5081 | 2x251 & 2x226 | 2x240 & 2x170 | 77 | 212,616 | 159 | 5,215,212 | JAAQYE01 |
| *P. lundensis* WS 5088 | 2x251 | 2x150 | 94 | 250,066 | 59 | 5,116,623 | JAAQYD01 |
| *P. lundensis* WS 5095 | 2x251 | 2x240 | 39 | 108,496 | 124 | 5,042,919 | JAAQYC01 |
| *P. lundensis* WS 5104 | 2x251 | 2x240 | 60 | 186,060 | 70 | 5,171,777 | JAAQYB01 |
| *P. lundensis* WS 5110 | 2x251 & 2x251 | 2x240 & 2x150 | 40 | 196,975 | 80 | 5,190,045 | JAAQYA01 |
| *P. lundensis* WS 5117 | 2x251 | 2x240 | 92 | 227,678 | 49 | 4,908,174 | JAAQXZ01 |
| *P. lundensis* WS 5118 | 2x251 | 2x240 | 123 | 159,291 | 86 | 5,091,333 | JAAQXY01 |
| *P. lundensis* WS 5119 | 2x251 | 2x240 | 132 | 184,907 | 95 | 5,409,286 | JAAQXX01 |
| *P. lundensis* WS 5121 | 2x251 | 2x240 | 125 | 325,341 | 58 | 5,007,675 | JAAQXW01 |
| *P. mandelii* WS 5114 | 2x226 & 2x251 | 2x170 & 2x150 | 68 | 690,265 | 35 | 6,818,060 | JAAQXV01 |
| *P. nitroreducens* DSM 14399 ^T^ | 2x251 | 2x240 | 138 | 855,214 | 50 | 6,171,316 | JAAQXU01 |
| *P. nitroreducens* WS 5012 | 2x251 | 2x240 | 100 | 449,658 | 43 | 6,757,787 | JAAQXT01 |
| *P. oryzihabitans* DSM 6835 ^T^ | 2x251 & 2x251 | 2x240 & 2x240 | 103 | 832,289 | 13 | 5,038,716 | JAAQXS01 |
| *P. oryzihabitans* WS 5017 | 2x251 | 2x150 | 60 | 1,068,312 | 19 | 4,995,228 | JAAQXR01 |
| *P. panacis* WS 4668 | 2x200 | 2x189 | 95 | 404,894 | 37 | 6,730,220 | JAARME01 |
| *P. peli* DSM 17833 ^T^ | 2x251 | 2x240 | 100 | 2,500,221 | 15 | 4,511,138 | JAAQXQ01 |
| *P. poae* WS 5103 | 2x251 | 2x230 | 30 | 322,592 | 41 | 5,528,914 | JAAQXP01 |
| *P. protegens* WS 5082 | 2x251 | 2x150 | 46 | 726,802 | 20 | 6,926,774 | JAARMD01 |
| *P. protegens* WS 5415 | 2x251 | 2x240 | 19 | 773,105 | 29 | 6,936,464 | JAAQXO01 |
| *P. proteolytica* WS 5031 | 2x251 | 2x230 | 67 | 274,032 | 47 | 5,912,257 | JAAQXN01 |
| *P. proteolytica* WS 5060 | 2x251 & 2x251 | 2x230 & 2x190 | 61 | 119,534 | 126 | 6,489,653 | JAAQXM01 |
| *P. proteolytica* WS 5126 | 2x251 | 2x220 | 53 | 145,597 | 85 | 6,136,959 | JAAQXL01 |
| *P. proteolytica* WS 5127 | 2x251 | 2x220 | 37 | 260,396 | 52 | 5,960,850 | JAAQXK01 |
| *P. proteolytica* WS 5128 | 2x251 | 2x220 | 45 | 287,694 | 44 | 5,951,709 | JAAQXJ01 |
| *P. proteolytica* WS 5147 | 2x251 & 2x251 | 2x240 & 2x220 | 90 | 108,148 | 129 | 6,421,398 | JAAQXI01 |
| *P. proteolytica* WS 5148 | 2x251 & 2x251 | 2x240 & 2x220 | 113 | 145,786 | 113 | 6,550,737 | JAAQXH01 |
| *P. psychrotolerans* DSM 15758 ^T^ | 2x251 | 2x240 | 48 | 324,503 | 40 | 6,012,013 | JAAQXG01 |
| *P. rhodesiae* WS 4669 | 2x200 | 2x189 | 117 | 219,939 | 65 | 5,983,759 | JAAQXF01 |
| *P. rhodesiae* WS 5107 | 2x251 | 2x150 | 67 | 224,025 | 58 | 5,961,510 | JAAQXE01 |
| *Pseudomonas* sp. 1 WS 5018 | 2x251 | 2x150 | 39 | 188,181 | 75 | 4,803,951 | JAAQXD01 |
| *Pseudomonas* sp. 2 WS 5013 | 2x226 | 2x170 | 78 | 710,455 | 10 | 4,453,840 | JAAQXC01 |
| *Pseudomonas* sp. 3 WS 5019 | 2x251 | 2x240 | 47 | 372,704 | 35 | 5,198,270 | JAAQXB01 |
| *Pseudomonas* sp. 4 WS 5011 | 2x251 | 2x240 | 100 | 216,844 | 88 | 4,604,264 | JAAQXA01 |
| *Pseudomonas* sp. 5 WS 5414 | 2x226 | 2x215 | 11 | 160,188 | 115 | 6,672,571 | JAAQWZ01 |
| *Pseudomonas* sp. 6 WS 5071 | 2x251 & 2x251 | 2x240 & 2x240 | 110 | 121,509 | 103 | 4,803,694 | JAAQWY01 |
| *Pseudomonas* sp. 6 WS 5078 | 2x251 | 2x150 | 96 | 264,864 | 41 | 4,738,897 | JAAQWX01 |
| *Pseudomonas* sp. 6 WS 5354 | 2x226 | 2x215 | 36 | 264,864 | 44 | 4,736,153 | JAAQWW01 |
| *Pseudomonas* sp. 7 WS 5051 | 2x251 | 2x240 | 66 | 345,925 | 38 | 5,142,615 | JAAQWV01 |
| *Pseudomonas* sp. 8 WS 5027 | 2x251 & 2x251 | 2x240 & 2x230 | 135 | 199,053 | 57 | 6,235,040 | JAAQWU01 |
| *Pseudomonas* sp. 8 WS 5086 | 2x251 & 2x226 | 2x190 & 2x170 | 59 | 198,962 | 61 | 6,148,335 | JAAQWT01 |
| *Pseudomonas* sp. 9 WS 5410 | 2x226 | 2x215 | 53 | 227,924 | 60 | 6,349,496 | JAAQWS01 |
| *Pseudomonas* sp. 9 WS 5411 | 2x226 | 2x215 | 28 | 197,246 | 70 | 5,861,914 | JAARMC01 |
| *Pseudomonas* sp. 9 WS 5412 | 2x251 | 2x240 | 41 | 223,471 | 71 | 6,171,677 | JAARMB01 |
| *Pseudomonas* sp. 9 WS 5413 | 2x251 | 2x240 | 50 | 194,584 | 85 | 6,421,091 | JAAQWR01 |
| *Pseudomonas* sp. 10 WS 5010 | 2x251 | 2x240 | 106 | 767,371 | 42 | 6,231,520 | JAAQWQ01 |
| *Pseudomonas* sp. 10 WS 5021 | 2x251 | 2x230 | 47 | 638,072 | 22 | 6,340,118 | JAAQWP01 |
| *Pseudomonas* sp. 10 WS 5059 | 2x251 | 2x230 | 32 | 620,406 | 28 | 6,195,332 | JAAQWO01 |
| *Pseudomonas* sp. 10 WS 5079 | 2x251 | 2x230 | 37 | 473,683 | 35 | 6,328,935 | JAAQWN01 |
| *Pseudomonas* sp. 10 WS 5111 | 2x251 & 2x251 | 2x190 & 2x190 | 52 | 201,692 | 75 | 6,318,683 | JAAQWM01 |
| *Pseudomonas* sp. 11 WS 5146 | 2x251 | 2x220 | 33 | 541,250 | 37 | 6,482,218 | JAAQWL01 |
| *Pseudomonas* sp. 11 WS 5532 | 2x251 & 2x251 | 2x240 & 2x220 | 49 | 285,395 | 69 | 7,371,843 | JAAQWK01 |
| *Pseudomonas* sp. 12 WS 5407 | 2x275 | 2x225 | 129 | 173,495 | 99 | 6,301,008 | JAAQWJ01 |
| *Pseudomonas* sp. 12 WS 5503 | 2x226 | 2x215 | 52 | 480,432 | 38 | 6,706,265 | JAAQWI01 |
| *Pseudomonas* sp. 13 WS 5406 | 2x275 | 2x200 | 76 | 305,182 | 43 | 6,400,167 | JAAQWH01 |
| *P. veronii* DSM 16272 | 2x200 | 2x189 | 113 | 101,893 | 208 | 7,901,729 | JAAQWG01 |
| *P. veronii* WS 4670 | 2x200 | 2x189 | 115 | 200,719 | 111 | 7,064,515 | JAAQWF01 |
| *P. veronii* WS 4671 | 2x200 | 2x189 | 102 | 161,010 | 125 | 7,122,147 | JAAQWE01 |
| *P. veronii* WS 5113 | 2x251 & 2x251 | 2x240 & 2x150 | 42 | 217,144 | 77 | 6,847,663 | JAAQWD01 |
| **^T^** – type strain |  |  |  |  |  |  |  |

**Supplementary Table 2.** *Pseudomonas* strains tested for proteolytic activity (agar diffusion assay)

| **strain** | **origin** |
| --- | --- |
| *P. aeruginosa* DSM 50071 ^T^ | unknown |
| *P. aeruginosa* WS 5022 | raw milk, Germany |
| *P. asplenii* DSM 17133 ^T^ | bird's nest fern *Asplenium nidus,* USA |
| *P. azotoformans* DSM 18862 ^T^ | paddies , Japan |
| *P. brenneri* DSM 15294 ^T^ | mineral water, France |
| *P. brenneri* WS 5091 | raw milk, Germany |
| *P. chengduensis* DSM 26382 ^T^ | waste disposal site, China |
| *P. chlororaphis* WS 5014 | raw milk, Germany |
| *P. chlororaphis* (ssp. *chlororaphis*) DSM 50083 ^T^ | plate contaminant, unkown |
| *P. corrugata* DSM 7228 ^T^ | *Lycopersicon esculentum,* UK |
| *P. deceptionensis* DSM 26521 ^T^ | marine sediment, Antarctica |
| *P. endophytica* B5 TT 44^T^ | stem tissue of *Solanum tuberosum*, Spain |
| *P. extremaustralis* DSM 17835 ^T^ | water pond, Antarctica |
| *P. fluorescens* DSM 50090 ^T^ | pre-filter tanks, UK |
| *P. fluorescens* WS 5037 | raw milk, Germany |
| *P. fragi* WS 5025 | raw milk, Germany |
| *P. fragi* DSM 3456 ^T^ | surface water, Germnay |
| *P. fragi* WS 5032 | raw milk, Germany |
| *P. fragi* WS 5045 | raw milk, Germany |
| *P. fragi* WS 5065 | raw milk, Germany |
| *P. fragi* WS 5087 | raw milk, Germany |
| *P. fragi* WS 5094 | raw milk, Germany |
| *P. fragi* WS 5102 | raw milk, Germany |
| *P. fragi* WS 5112 | raw milk, Germany |
| *P. fragi* WS 5120 | raw milk, Germany |
| *P. fragi* WS 5123 | raw milk, Germany |
| *P. fragi* WS 5124 | water, Germany |
| *P. fragi* WS 5125 | water, Germany |
| *P. fragi* WS 5416 | raw milk, Germany |
| *P. gessardii* DSM 17152 ^T^ | mineral water, France |
| *P. gessardii* WS 5049 | raw milk, Germany |
| *P. gessardii* WS 5408 | raw milk, Germany |
| *P. grimontii* DSM 17515 ^T^ | mineral water, France |
| *P. ‘haemolytica‘* DSM 108988 | raw milk, Germany |
| *P. ‘haemolytica‘* DSM 108987^T^ | raw milk, Germany |
| *P. helleri* DSM 29165^T^ | raw milk, Germany |
| *P. helleri* DSM 28141 | raw milk, Germany |
| *P. kilonensis* DSM 13647 ^T^ | agricultural soil, Germany |
| *P. koreensis* DSM 16610 ^T^ | agricultural soil, Korea |
| *P. koreensis* WS 5015 | raw milk, Germany |
| *P. lactis* DSM 29167 ^T^ | raw milk, Germany |
| *P. lactis* WS 4997 | raw milk, Germany |
| *P. lactis* WS 5000 | raw milk, Germany |
| *P. lactis* WS 5404 | raw milk, Germany |
| *P. lactis* WS 5405 | raw milk, Germany |
| *P. libanensis* DSM 17149 ^T^ | spring water, Lebanon |
| *P. lini* DSM 16768 ^T^ | rhizospheric soil, France |
| *P. lundensis* DSM 6252 ^T^ | prepacked beef, Sweden |
| *P. lundensis* WS 5024 | raw milk, Germany |
| *P. lundensis* WS 5026 | raw milk, Germany |
| *P. lundensis* WS 5081 | raw milk, Germany |
| *P. lundensis* WS 5088 | raw milk, Germany |
| *P. lundensis* WS 5095 | raw milk, Germany |
| *P. lundensis* WS 5104 | raw milk, Germany |
| *P. lundensis* WS 5110 | raw milk, Germany |
| *P. lundensis* WS 5117 | raw milk, Germany |
| *P. lundensis* WS 5118 | raw milk, Germany |
| *P. lundensis* WS 5119 | raw milk, Germany |
| *P. lundensis* WS 5121 | Tetra Pak, Germany |
| *P. mandelii* DSM 17967 ^T^ | mineral water, France |
| *P. mandelii* WS 5114 | raw milk, Germany |
| *P. marginalis* DSM 13124 ^T^ | *Cichorium intybus,* USA |
| *P. mosselii* DSM 17497 ^T^ | medical specimen, France |
| *P. nitroreducens* DSM 14399 ^T^ | oil-brine, Japan |
| *P. nitroreducens* WS 5012 | unknown, Germany |
| *P. orientalis* DSM 17489 ^T^ | spring water, Lebanon |
| *P. oryzihabitans* DSM 6835 ^T^ | rice paddy, Japan |
| *P. oryzihabitans* WS 5017 | unknown, Germany |
| *P. panacis* WS 4668 | raw milk, Germany |
| *P. panacis* DSM 18529 ^T^ | rusty root lesions of Korean ginseng, South Korea |
| *P. paralactis* DSM 29164 ^T^ | raw milk, Germany |
| *P. peli* DSM 17833 ^T^ | nitrifying enrichment culture, Belgium |
| *P. poae* DSM 14936 ^T^ | phyllosphere of grasses, Germany |
| *P. poae* WS 5103 | raw milk, Germany |
| *P. protegens* CHA0^T^ | soil suppressing black root rot of tobacco, Switzerland |
| *P. protegens* WS 5082 | raw milk, Germany |
| *P. proteolytica* DSM 15321 ^T^ | cyanobacterial mat samples, Antarctica |
| *P. proteolytica* WS 5031 | raw milk, Germany |
| *P. proteolytica* WS 5060 | raw milk, Germany |
| *P. proteolytica* WS 5126 | raw milk, Germany |
| *P. proteolytica* WS 5127 | raw milk, Germany |
| *P. proteolytica* WS 5128 | raw milk, Germany |
| *P. proteolytica* WS 5147 | brown horseraddish, Germany |
| *P. proteolytica* WS 5148 | water, Germany |
| *P. psychrophila* DSM 17535 ^T^ | coldroom for food storage, Japan |
| *P. psychrotolerans* DSM 15758 ^T^ | water under a dog's cage in a Medical Clinic for Small Animals, Austria |
| *P. rhodesiae* DSM 14020 ^T^ | mineral water, France |
| *P. rhodesiae* WS 4669 | cow, Germany |
| *P. rhodesiae* WS 5107 | raw milk, Germany |
| *P. ‘saxonica‘* DSM 108989 ^T^ | raw milk, Germany |
| *P. ‘saxonica‘* DSM 108990 | raw milk, Germany |
| *P. soli* DSM 28043 ^T^ | soil, Spain |
| *Pseudomonas* sp. 1 WS 5018 | raw milk, Germany |
| *Pseudomonas* sp. 2 WS 5013 | semi-finished milk product, Germany |
| *Pseudomonas* sp. 3 WS 5019 | water, Germany |
| *Pseudomonas* sp. 4 WS 5011 | raw milk, Germany |
| *Pseudomonas* sp. 5 WS 5414 | raw milk, Germany |
| *Pseudomonas* sp. 6 WS 5071 | raw milk, Germany |
| *Pseudomonas* sp. 6 WS 5078 | raw milk, Germany |
| *Pseudomonas* sp. 6 WS 5354 | raw milk, Germany |
| *Pseudomonas* sp. 7 WS 5051 | raw milk, Germany |
| *Pseudomonas* sp. 8 WS 5027 | raw milk, Germany |
| *Pseudomonas* sp. 8 WS 5086 | raw milk, Germany |
| *Pseudomonas* sp. 9 WS 5410 | raw milk, Germany |
| *Pseudomonas* sp. 9 WS 5411 | raw milk, Germany |
| *Pseudomonas* sp. 9 WS 5412 | raw milk, Germany |
| *Pseudomonas* sp. 10 WS 5010 | raw milk, Germany |
| *Pseudomonas* sp. 10 WS 5021 | raw milk, Germany |
| *Pseudomonas* sp. 10 WS 5059 | raw milk, Germany |
| *Pseudomonas* sp. 10 WS 5079 | raw milk, Germany |
| *Pseudomonas* sp. 10 WS 5111 | raw milk, Germany |
| *Pseudomonas* sp. 11 WS 5532 | unknown |
| *Pseudomonas* sp. 11 WS 5146 | milk product, Germany |
| *Pseudomonas* sp. 12 DSM 29142 | raw milk, Germany |
| *Pseudomonas* sp. 12 WS 5407 | raw milk, Germany |
| *Pseudomonas* sp. 12 WS 5503 | raw milk, Germany |
| *Pseudomonas* sp. 13 WS 5406 | raw milk, Germany |
| *P. synxantha* DSM 18928 ^T^ | cream, USA |
| *P. syringae subsp. syringae* DSM 10604 ^T^ | *Syringa vulgaris*, UK |
| *P. taetrolens* DSM 21104 ^T^ | musty egg, unknown |
| *P. trivialis*  DSM 14937 ^T^ | phyllosphere of grasses, Germnay |
| *P. veronii* DSM 11331 ^T^ | mineral water, France |
| *P. veronii* DSM 16272 | sediment, Netherlands |
| *P. veronii* WS 4670 ^T^ | semi-finished milk product, Germany |
| *P. veronii* WS 4671 | semi-finished milk product, Germany |
| *P. veronii* WS 5113 | raw milk, Germany |
| *P. viridiflava* DSM 11124 ^T^ | dwarf or runner bean, Switzerland |
| *P. weihenstephanensis* DSM 29140 | raw milk, Germany |
| *P. weihenstephanensis* DSM 29166 ^T^ | raw milk, Germany |
| **^T^** – type strain | |

**Supplementary Table 3.** Genome data of *Pseudomonas* type strains downloaded from NCBI

| **strain** | **NCBI WGS ID** | **RefSeq/GenBank IDs** |
| --- | --- | --- |
| *P. abietaniphila* ATCC 700689^T^ | FNCO01 |  |
| *P. abyssi* MT 5 ^T^ | NTMR01 |  |
| *P. aeruginosa* DSM 50071 ^T^ | JYLC01 |  |
| *P. aestusnigri* CECT 8317 ^T^ | FNVE01 |  |
| *P. alcaligenes* NBRC 14159 ^T^ | BATI01 |  |
| *P. alcaliphila* JCM 10630 ^T^ | FNAE01 |  |
| *P. alkylphenolica* KL28 ^T^ |  | NZ_CP009048.1 |
| *P. amygdali* ICMP 3918 ^T^ | LJPQ01 |  |
| *P. anguilliseptica* DSM 12111 ^T^ | FNSC01 |  |
| *P. antarctica* DSM 15318 ^T^ | UYXQ01 |  |
| *P. argentinensis* LMG 22563 ^T^ | FORC01 |  |
| *P. arsenicoxydans* CECT 7543 ^T^ |  | LT629705.1 |
| *P. 'asiatica'* RYU5 ^T^ |  | DRR138841 (SRA) |
| *P. asplenii* ATCC 23835 ^T^ |  | LT629777.1 |
| *P. asturiensis* LMG 26898 ^T^ | FRDA01 |  |
| *P. avellanae* BPIC 631 ^T^ | AKBS01 |  |
| *P. azotifigens* DSM 17556 ^T^ | AUDU01 |  |
| *P. azotoformans* LMG 21611 ^T^ |  | NZ_LT629702.1 |
| *P. baetica* LMG 25716 ^T^ | PHHE01 |  |
| *P. balearica* DSM 6083 ^T^ |  | CP007511.1 |
| *P. bauzanensis* DSM 22558 ^T^ | FOGN01 |  |
| *P. benzenivorans* DSM 8628 ^T^ | FNCT01 |  |
| *P. 'bohemica'* IA19 ^T^ | NKHL01 |  |
| *P. borbori* DSM 17834 ^T^ | FOWX01 |  |
| *P. brenneri* DSM 15294 ^T^ | VFIL01 |  |
| *P. caeni* DSM 24390 ^T^ | ATXQ01 |  |
| *P. canadensis* 2-92 ^T^ | AYTD01 |  |
| *P. cannabina* ICMP 2823 ^T^ | FNKU01 |  |
| *P. caricapapayae* ICMP 2855 ^T^ | LJPW01 |  |
| *P. caspiana* FBF102 ^T^ | LOHF01 |  |
| *P. cedrina* ssp. *cedrina* DSM 17516 ^T^ | MNPW01 |  |
| *P. cerasi* 58 ^T^ |  | NZ_LT222313.1 - NZ_LT222319.1 |
| *P. chlororaphis* ssp. *aurantiaca* LMG 21630 ^T^ |  | NZ_LT629747.1 |
| *P. chlororaphis* ssp. *aureofaciens* NBRC 3521 ^T^ | BBQB01 |  |
| *P. chlororaphis* ssp. *chlororaphis* DSM 50083 ^T^ | VFIN01 |  |
| *P. chlororaphis* ssp. *piscium* DSM 21509 ^T^ | LHUZ01 |  |
| *P. cichorii* ATCC 10857 ^T^ | FNIK01 |  |
| *P. citronellolis* LMG 18378 ^T^ | FOLS01 |  |
| *P. coleopterorum* LMG 28558 ^T^ | FNTZ01 |  |
| *P. composti* CCUG 59231 ^T^ | FOWP01 |  |
| *P. congelans* DSM 14939 ^T^ | FNJH01 |  |
| *P. corrugata* DSM 7228 ^T^ | LHVK01 |  |
| *P. costantinii* LMG 22119 ^T^ | MDDR01 |  |
| *P. cremoricolorata* DSM 17059 ^T^ | AUEA01 |  |
| *P. cuatrocienegasensis* CIP 109853 ^T^ | FOFP01 |  |
| *P. deceptionensis* DSM 26521 ^T^ | JYKX01 |  |
| *P. delhiensis* CCM 7361 ^T^ | FNEC01 |  |
| *P. donghuensis* HYS ^T^ | AJJP01 |  |
| *P. endophytica* BSTT44 ^T^ | LLWH01 |  |
| *P. entomophila* L48 ^T^ |  | NC_008027.1 |
| *P. extremaustralis* DSM 17835 ^T^ | VFET01 |  |
| *P. extremorientalis* LMG 19695 ^T^ | MDGK01 |  |
| *P. ficuserectae* ICMP 7848 ^T^ | LJQJ01 |  |
| *P. flavescens* NBRC 103044 ^T^ | BCZZ01 |  |
| *P. flexibilis* CGMCC 1.1365 ^T^ | FMUP01 |  |
| *P. floridensis* GEV388 ^T^ | MUIO01 |  |
| *P. fluorescens* DSM 50090 ^T^ | VFEP01 |  |
| *P. fluvialis* ASS-1 ^T^ | NMQV01 |  |
| *P. formosensis* JCM 18415 ^T^ | FOYD01 |  |
| *P. fragi* B25 ^T^ | AHZX01 |  |
| *P. fulva* NBRC 16637 ^T^ | JHYU01 |  |
| *P. furukawaii* KF707 ^T^ | AJMR01 |  |
| *P. fuscovaginae* LMG 2158 ^T^ |  | NZ_LT629972.1 |
| *P. 'gallaeciensis'* V113 ^T^ | LMAZ01 |  |
| *P. gessardii* DSM 17152 ^T^ | VFEW01 |  |
| *P. 'qingdaonensis'* JJ3 ^T^ | PHTD01 |  |
| *P. graminis* DSM 11363 ^T^ | FOHW01 |  |
| *P. granadensis* LMG 27940 ^T^ |  | LT629778.1 |
| *P. grimontii* DSM 17515 ^T^ | VFES01 |  |
| *P. guangdongensis* CCTCC 2012022 ^T^ |  | LT629780.1 |
| *P. guariconensis* LMG 27394 ^T^ | FMYX01 |  |
| *P. guguanensis* JCM 18416 ^T^ | FNJJ01 |  |
| *P. guineae* LMG 24016 ^T^ | FOQL01 |  |
| *P. 'haemolytica'* DSM 108987 ^T^ | VOIW01 |  |
| *P. ‘haemolytica’* DSM 108988 | VOIX01 |  |
| *P. helleri* DSM 29165 ^T^ | JYLD01 |  |
| *P. helleri* DSM 28141 | JYLE01 |  |
| *P. hussainii* JCM 19513 ^T^ | FOAS01 |  |
| *P. indica* NBRC 103045 ^T^ | BDAC01 |  |
| *P. indoloxydans* JCM 14246 ^T^ | QASO01 |  |
| *P. japonica* NBRC 103040 ^T^ | BBIR01 |  |
| *P. jessenii* DSM 17150 ^T^ | NIWT01 |  |
| *P. jinjuensis* NBRC 103047 ^T^ | BDAD01 |  |
| *P. kilonensis* DSM 13647 ^T^ | LHVH01 |  |
| *P. knackmussii* B13 ^T^ |  | HG322950.1 |
| *P. 'kribbensis'* 46-2 ^T^ |  | NZ_CP029608.1 |
| *P. kunmingensis* DSM 25974 ^T^ | FORS01 |  |
| *P. kuykendallii* NRRL B-59562 ^T^ | FNNU01 |  |
| *P. lactis* DSM 29167 ^T^ | JYLO01 |  |
| *P. 'laurylsulfatovorans'* AP3_22 ^T^ | MUJK01 |  |
| *P. libanensis* DSM 17149 ^T^ | JYLH01 |  |
| *P. lini* DSM 16768 ^T^ | JYLB01 |  |
| *P. linyingensis* LMG 25967 ^T^ | FNZE01 |  |
| *P. litoralis* 2SM5 ^T^ | LT629748.1 |  |
| *P. lundensis* DSM 6252 ^T^ | JYKY01 |  |
| *P. lurida* LMG 21995 ^T^ | PDJB01 |  |
| *P. lutea* DSM 17257 ^T^ | JRMB01 |  |
| *P. luteola* NBRC 103146 ^T^ | BDAE01 |  |
| *P. mandelii* DSM 17967 ^T^ | VFIM01 |  |
| *P. 'mangrovi'* TC11 ^T^ | QASN01 |  |
| *P. marginalis* DSM 13124 ^T^ | VFEQ01 |  |
| *P. marincola* JCM 14761 ^T^ | FPBC01 |  |
| *P. 'massiliensis'* CB1 ^T^ | CCYK01 |  |
| *P. mediterranea* CFBP 5447 ^T^ | AUPB01 |  |
| *P. meliae* CFBP 3225 ^T^ | JYHE01 |  |
| *P. mendocina* NBRC 14162 ^T^ | BBQC01 |  |
| *P. migulae* NBRC 103157 ^T^ | BDAG01 |  |
| *P. mohnii* DSM 18327 ^T^ | FNRV01 |  |
| *P. monteilii* NBRC 103158 ^T^ | JHYV01 |  |
| *P. mosselii* DSM 17497 ^T^ | JHYW01 |  |
| *P. mucidolens* NBRC 103159 ^T^ | BDAH01 |  |
| *P. oceani* DSM 100277 ^T^ | PPSK01 |  |
| *P. oleovorans* ssp. *lubricantis* RS1 ^T^ | FNZC01 |  |
| *P. oleovorans* ssp. *oleovorans* NBRC 13583 ^T^ | BDAL01 |  |
| *P. orientalis* DSM 17489 ^T^ | JYLM01 |  |
| *P. 'oryzae'* KCTC 32247 ^T^ |  | LT629751.1 |
| *P. otitidis* DSM 17224 ^T^ | FOJP01 |  |
| *P. pachastrellae* CCUG 46540 ^T^ | MUBC01 |  |
| *P. palleroniana* LMG 23076 ^T^ | PYWX01 |  |
| *P. panacis* DSM 18529 ^T^ | VFER01 |  |
| *P. panipatensis* CCM 7469 ^T^ | FNDS01 |  |
| *P. parafulva* DSM 17004 ^T^ | AUEB01 |  |
| *P. paralactis* DSM 29164 ^T^ | JYLN01 |  |
| *P. pelagia* CL-AP6 ^T^ | AROI01 |  |
| *P. 'pharmafabricae'* ZYSR67-Z ^T^ | PIYS01 |  |
| *P. plecoglossicida* NBRC 103162 ^T^ | JHYX01 |  |
| *P. poae* DSM 14936 ^T^ | JYLI01 |  |
| *P. pohangensis* DSM 17875 ^T^ |  | LT629785.1 |
| *P. prosekii* LMG 26867 ^T^ |  | LT629762.1 |
| *P. protegens* CHA0 ^T^ |  | NC_021237.1 |
| *P. proteolytica* DSM 15321 ^T^ | VFEV01 |  |
| *P. psychrophila* DSM 17535 ^T^ | JYKZ01 |  |
| *P. punonensis* CECT 8089 ^T^ | FRBQ01 |  |
| *P. putida* NBRC 14164 ^T^ |  | NC_021505.1 |
| *P. reidholzensis* CCOS 865 ^T^ | UNOZ01 |  |
| *P. reinekei* MT1 ^T^ | MSTQ01 |  |
| *P. resinovorans* DSM 21078 ^T^ | AUIE01 |  |
| *P. rhizosphaerae* DSM 16299 ^T^ |  | CP009533.1 |
| *P. rhodesiae* DSM 14020 ^T^ | VFEU01 |  |
| *P. sabulinigri* JCM 14963 ^T^ |  | LT629763.1 |
| *P. sagittaria* JCM 18195 ^T^ | FOXM01 |  |
| *P. 'saxonica'* DSM 108989 ^T^ | VFIO01 |  |
| *P. ‘saxonica’* DSM 108990 | VFIP01 |  |
| *P. salegens* CECT 8338 ^T^ |  | LT629787.1 |
| *P. salomonii* ICMP 14252 ^T^ | FNOX01 |  |
| *P. saponiphila* DSM 9751 ^T^ | FNTJ01 |  |
| *P. savastanoi* ICMP 4352 ^T^ | LJRJ01 |  |
| *P. 'sediminis'* PI11 ^T^ | NIQU01 |  |
| *P. segetis* CIP 108523 ^T^ | FZOG01 |  |
| *P. seleniipraecipitans* LMG 25475 ^T^ | FNBM01 |  |
| *P. 'sichuanensis'* WCHPs060039 ^T^ | QKVM01 |  |
| *P. 'sihuiensis'* KCTC 32246 ^T^ |  | LT629797.1 |
| *P. silesiensis* A3 ^T^ |  | NZ_CP014870.1 |
| *P. simiae* CCUG 50988 ^T^ | FOKB01 |  |
| *P. soli* LMG 27941 ^T^ | FOEQ01 |  |
| *P. 'songnenensis'* NEAU-ST5-5 ^T^ | RFFN01 |  |
| *Pseudomonas* sp. DSM 29142 | JYLP01 |  |
| *P. straminea* JCM 2783 ^T^ | FOMO01 |  |
| *P. stutzeri* ATCC 17588 ^T^ |  | NC_015740.1 |
| *P. synxantha* DSM 18928 ^T^ | JYLJ01 |  |
| *P. syringae* ssp. *syringae* DSM 10604 ^T^ | JALK01 |  |
| *P. taeanensis* MS-3 ^T^ | AWSQ01 |  |
| *P. taetrolens* DSM 21104 ^T^ | JYLA01 |  |
| *P. taiwanensis* DSM 21245 ^T^ | AUEC01 |  |
| *P. thermotolerans* DSM 14292 ^T^ | AQPA01 |  |
| *P. thivervalensis* DSM 13194 ^T^ | LHVE01 |  |
| *P. tolaasii* NCPPB 2192 ^T^ |  | NZ_CP020369.1 |
| *P. toyotomiensis* JCM 15604 ^T^ | FOXK01 |  |
| *P. tremae* ICMP 9151 ^T^ | LJRO01 |  |
| *P. trivialis* DSM 14937 ^T^ | JYLK01 |  |
| *P. tuomuerensis* JCM 14085 ^T^ | JTAK01 |  |
| *P. umsongensis* DSM 16611 ^T^ | NIWU01 |  |
| *P. veronii* DSM 11331 ^T^ | JYLL01 |  |
| *P. versuta* L10.10 ^T^ |  | NZ_CP012676.1 |
| *P. viridiflava* DSM 6694 ^T^ | JRXH01 |  |
| *P. vranovensis* DSM 16006 ^T^ | AUED01 |  |
| *P. wadenswilerensis* CCOS 864 ^T^ | UIDD01 |  |
| *P. weihenstephanensis* DSM 29166 ^T^ | JYLF01 |  |
| *P. weihenstephanensis* DSM 29140 | JYLG01 |  |
| *P. xanthomarina* DSM 18231 ^T^ | FQXA01 |  |
| *P. xinjiangensis* NRRL B-51270 ^T^ |  | NZ_LT629736.1 |
| *P. yamanorum* LMG 27247 ^T^ |  | LT629793.1 |
| *P. zeshuii* KACC 15471 ^T^ | FQYS01 |  |
| *P. zhaodongensis* NEAU-ST5-21 ^T^ | RFFM01 |  |
| ^T^ – type strain |  |  |

**Supplementary Table 4.** Shapiro-Wilk and Levene tests on agar diffusion assay data

| **statistical test** | **p-values** | | |
| --- | --- | --- | --- |
|  | **day 3** | **day 4** | **day 7** |
| Levene’s test  (homogeneity of variance) | 0.5989 | 0.3050 | 0.1456 |
| Shapiro-Wilk test  (normality of data) | 0.7732 | 0.6371 | 0.2840 |

**Supplementary Table 5.** Validly described *Pseudomonas* species/subspecies (LPSN as of March 2019)

| **species**  **number** | **species/subspecies (reference^LPSN^)** | **species**  **number** | **species/subspecies (reference^LPSN^)** |
| --- | --- | --- | --- |
| 1 | *P. abietaniphila* (Mohn *et al.*1999) | 93 | *P. litoralis* (Pascual *et al.* 2012) |
| 2 | *P. abyssi* (Wei *et al.* 2018) | 94 | *P. lundensis* (Molin *et al.* 1986) |
| 3 | *P. aeruginosa* (Schroeter 1872) | 95 | *P. lurida* (Behrendt *et al.* 2007) |
| 4 | *P. aestusnigri* (Sanchez *et al.* 2014) | 96 | *P. lutea* (Peix *et al.* 2004) |
| 5 | *P. agarici* (Young 1970) | 97 | *P. luteola* (Kodama *et al.* 1985) |
| 6 | *P. alcaligenes* (Monias 1928) | 98 | *P. mandelii* (Verhille *et al.* 1999) |
| 7 | *P. alcaliphila* (Yumoto *et al.* 2001) | 99 | *P. marginalis* (Brown 1918) |
| 8 | *P. alkylphenolica* (Mulet *et al.* 2015) | 100 | *P. marincola* (Romanenko *et al.* 2008) |
| 9 | *P. amygdali* (Psallidas & Panagopoulos 1975) | 101 | *P. matsuisoli* (Lin *et al.* 2015) |
| 10 | *P. anguilliseptica* (Wakabayashi & Egusa 1972) | 102 | *P. mediterranea* (Catara *et al.* 2002) |
| 11 | *P. antarctica* (Reddy *et al.* 2004) | 103 | *P. meliae* (Ogimi 1981) |
| 12 | *P. argentinensis* (Peix *et al.* 2005) | 104 | *P. mendocina* (Palleroni 1970) |
| 13 | *P. arsenicoxydans* (Campos *et al.* 2011) | 105 | *P. meridian* (Reddy *et al.* 2004) |
| 14 | *P. asplenii* (Ark & Tompkins 1946) | 106 | *P. migulae* (Verhille *et al.* 1999) |
| 15 | *P. asturiensis* (González *et al.* 2013) | 107 | *P. mohnii* (Cámara *et al.* 2007) |
| 16 | *P. asuensis* (Reddy & Garcia-Pichel 2015) | 108 | *P. monteilii* (Elomari *et al.* 1997) |
| 17 | *P. avellanae* (Janse *et al.* 1997) | 109 | *P. moorei* (Cámara *et al.* 2007) |
| 18 | *P. azotifigens* (Hatayama *et al.* 2005) | 110 | *P. moraviensis* (Tvrzová *et al.* 2006) |
| 19 | *P. azotoformans* (Iizuka & Komagata 1963) | 111 | *P. mosselii* (Dabboussi *et al.* 2002) |
| 20 | *P. baetica* (López *et al.* 2012) | 112 | *P. mucidolens* (Levine & Anderson 1932) |
| 21 | *P. balearica* (Bennasar *et al.* 1996) | 113 | *P. nitritireducens* (Wang *et al.* 2015) |
| 22 | *P. bauzanensis* (Zhang *et al.* 2011) | 114 | *P. nitroreducens* (Iizuka & Komagata 1964) |
| 23 | *P. benzenivorans* (Lang *et al.* 2012) | 115 | *P. oceani* (Wang & Sun 2016) |
| 24 | *P. borbori* (Vanparys *et al.* 2006) | 116 | *P. oleovorans* ssp. *lubricantis* (Saha *et al.* 2010) |
| 25 | *P. brassicacearum ssp. brassicacearum* (Achouak *et al.* 2000) | 116 | *P. oleovorans* ssp. *oleovorans* (Lee & Chandler 1941) |
| 25 | *P. brassicacearum ssp. neoaurantiaca* (Ivanova *et al.* 2009*)* | 117 | *P. orientalis* (Dabboussi *et al.* 2002) |
| 26 | *P. brenneri* (Baïda *et al.* 2002) | 118 | *P. oryzihabitans* (Kodama *et al.* 1985) |
| 27 | *P. caeni* (Xiao *et al.* 2009) | 119 | *P. otitidis* (Clark *et al.* 2006) |
| 28 | *P. canadensis* (Tambong *et al.* 2017) | 120 | *P. pachastrellae* (Romanenko *et al.* 2005) |
| 29 | *P. cannabina* (ex Šutič & Dowson 1959) | 121 | *P. palleroniana* (Gardan *et al.* 2002) |
| 30 | *P. caricapapayae* (Robbs 1956) | 122 | *P. panacis* (Park *et al.* 2005) |
| 31 | *P. caspiana* (Busquets *et al.* 2017) | 123 | *P. panipatensis* (Gupta *et al.* 2008) |
| 32 | *P. cedrina* ssp*. cedrina* (Dabboussi *et al.* 2002) | 124 | *P. parafulva* (Uchino *et al.* 2002) |
| 32 | *P. cedrina* ssp*. fulgida* (Behrendt *et al.* 2009) | 125 | *P. paralactis* (von Neubeck *et al.* 2017) |
| 33 | *P. cerasi* (Kaluzna *et al.* 2017) | 126 | *P. pelagia* (Hwang *et al.* 2009) |
| 34 | *P. chengduensis* (Tao *et al.* 2014) | 127 | *P. peli* (Vanparys *et al.* 2006) |
| 35 | *P. chlororaphis* ssp*. aurantiaca* (Nakhimovskaya 1948) | 128 | *P. pertucinogena* (Kawai & Yabuuchi 1975) |
| 35 | *P. chlororaphis* ssp*. aureofaciens* (Kluyver 1956) | 129 | *P. plecoglossicida* (Nishimori *et al.* 2000) |
| 35 | *P. chlororaphis* ssp*. chlororaphis* (Guignard & Sauvageau 1894) | 130 | *P. poae* (Behrendt *et al.* 2003) |
| 35 | *P. chlororaphis* ssp*. piscium* (Burr *et al.* 2010) | 131 | *P. pohangensis* (Weon *et al.* 2006) |
| 36 | *P. cichorii* (Swingle 1925) | 132 | *P. populi* (Anwar *et al.* 2016) |
| 37 | *P. cissicola* (Takimoto 1939) | 133 | *P. profundi* (Sun *et al.* 2018) |
| 38 | *P. citronellolis* (Seubert 1960) | 134 | *P. prosekii* (Kosina *et al.* 2014) |
| 39 | *P. coleopterorum* (Menéndez *et al.* 2015) | 135 | *P. protegens* (Ramette *et al.* 2012) |
| 40 | *P. composti* (Gibello *et al.* 2011) | 136 | *P. proteolytica* (Reddy *et al.* 2004) |
| 41 | *P. congelans* (Behrendt *et al.* 2003) | 137 | *P. psychrophila* (Yumoto *et al.* 2002) |
| 42 | *P. corrugata* (Roberts & Scarlett 1981) | 138 | *P. psychrotolerans* (Hauser *et al.* 2004) |
| 43 | *P. costantinii* (Munsch *et al.* 2002) | 139 | *P. punonensis* (Ramos *et al.* 2013) |
| 44 | *P. cremoricolorata* (Uchino *et al.* 2002) | 140 | *P. putida* (Trevisan 1889) |
| 45 | *P. cuatrocienegasensis* (Escalante *et al.* 2009) | 141 | *P. reidholzensis* (Frasson *et al.* 2017) |
| 46 | *P. deceptionensis* (Carrión *et al.* 2011) | 142 | *P. reinekei* (Cámara *et al.* 2007) |
| 47 | *P. delhiensis* (Prakash *et al.* 2007) | 143 | *P. resinovorans* (Delaporte *et al.* 1961) |
| 48 | *P. donghuensis* (Gao *et al.* 2015) | 144 | *P. rhizosphaerae* (Peix *et al.* 2003) |
| 49 | *P. duriflava* (Liu *et al.* 2008) | 145 | *P. rhodesiae* (Coroler *et al.* 1997) |
| 50 | *P. endophytica* (Ramírez-Bahena *et al.* 2015) | 146 | *P. sabulinigri* (Kim *et al.* 2009) |
| 51 | *P. entomophila* (Mulet *et al.* 2012) | 147 | *P. sagittaria* (Lin *et al.* 2013) |
| 52 | *P. extremaustralis* (López *et al.* 2010) | 148 | *P. salegens* (Amoozegar *et al.* 2014) |
| 53 | *P. extremorientalis* (Ivanova *et al.* 2002) | 149 | *P. salina* (Zhong *et al.* 2015) |
| 54 | *P. ficuserectae* (Goto 1983) | 150 | *P. salomonii* (Gardan *et al.* 2002) |
| 55 | *P. flavescens* (Hildebrand *et al.* 1994) | 151 | *P. saponiphila* (Lang *et al.* 2012) |
| 56 | *P. flexibilis* (Shin *et al.* 2016) | 152 | *P. savastanoi* (Janse 1982) |
| 57 | *P. floridensis* (Timilsina *et al.* 2018) | 153 | *P. segetis* (Park *et al.* 2006) |
| 58 | *P. fluorescens* (Migula 1895) | 154 | *P. seleniipraecipitans* (Hunter & Manter 2011) |
| 59 | *P. fluvialis* (Sudan *et al.* 2018) | 155 | *P. sesami* (Madhaiyan *et al.* 2017) |
| 60 | *P. formosensis* (Lin *et al.* 2013) | 156 | *P. silesiensis* (Kaminski *et al.* 2018) |
| 61 | *P. fragi* (Gruber 1905) | 157 | *P. simiae* (Vela *et al.* 2006) |
| 62 | *P. frederiksbergensis* (Andersen *et al.* 2000) | 158 | *P. soli* (Pascual *et al.* 2015) |
| 63 | *P. fulva* (Iizuka & Komagata 1963) | 159 | *P. straminea* (Iizuka & Komagata 1963) |
| 64 | *P. furukawaii* (Kimura *et al.* 2018) | 160 | *P. stutzeri* (Lehmann & Neumann 1896 |
| 65 | *P. fuscovaginae (ex* Tanii *et al.* 1976) | 161 | *P. synxantha* (Ehrenberg 1840) |
| 66 | *P. gelidicola* (Kadota 1951) | 162 | *P. syringae* ssp*. syringae* (van Hall 1902) |
| 67 | *P. gessardii* (Verhille *et al.* 1999) | 163 | *P. taeanensis* (Lee *et al.* 2010) |
| 68 | *P. glareae* (Romanenko *et al.* 2015) | 164 | *P. taetrolens* (Haynes 1957) |
| 69 | *P. graminis* (Behrendt *et al.* 1999) | 165 | *P. taiwanensis* (Wang *et al.* 2010) |
| 70 | *P. granadensis* (Pascual *et al.* 2015) | 166 | *P. tarimensis* (Anwar *et al.* 2017) |
| 71 | *P. grimontii* (Baïda *et al.* 2002) | 167 | *P. thermotolerans* (Manaia & Moore 2002) |
| 72 | *P. guangdongensis* (Yang *et al.* 2013) | 168 | *P. thivervalensis* (Achouak *et al.* 2000) |
| 73 | *P. guariconensis* (Toro *et al.* 2013) | 169 | *P. tolaasii* (Paine 1919) |
| 74 | *P. guguanensis* (Liu *et al.* 2013) | 170 | *P. toyotomiensis* (Hirota *et al.* 2011) |
| 75 | *P. guineae* (Bozal *et al.* 2007) | 171 | *P. tremae* (Gardan *et al.* 1999) |
| 76 | *P. helleri* (von Neubeck *et al.* 2016) | 172 | *P. trivialis* (Behrendt *et al.* 2003) |
| 77 | *P. helmanticensis* (Ramírez *et al.* 2014) | 173 | *P. tuomuerensis* (Xin *et al.* 2009) |
| 78 | *P. hussainii* (Hameed *et al.* 2014) | 174 | *P. turukhanskensis* (Korshunova *et al.* 2016) |
| 79 | *P. indica* (Pandey *et al.* 2002) | 175 | *P. umsongensis* (Kwon *et al.* 2003) |
| 80 | *P. indoloxydans* (Gray 1928) | 176 | *P. vancouverensis* (Mohn *et al.* 1999) |
| 81 | *P. japonica* (Pungrasmi *et al.* 2008) | 177 | *P. veronii* (Elomari *et al.* 1996) |
| 82 | *P. jessenii* (Verhille *et al.* 1999) | 178 | *P. versuta* (See *et al.* 2017) |
| 83 | *P. jinjuensis* (Kwon *et al.* 2003) | 179 | *P. viridiflava* (Burkholder 1930) |
| 84 | *P. kilonensis* (Sikorski *et al.* 2001) | 180 | *P. vranovensis* (Tvrzová *et al.* 2006) |
| 85 | *P. knackmussii* (Stolz *et al.* 2007) | 181 | *P. wadenswilerensis* (Frasson *et al.* 2017) |
| 86 | *P. koreensis* (Kwon *et al.* 2003) | 182 | *P. weihenstephanensis* (von Neubeck *et al.* 2016) |
| 87 | *P. kunmingensis* (Xie *et al.* 2014) | 183 | *P. xanthomarina* (Romanenko *et al.* 2005) |
| 88 | *P. kuykendallii* (Hunter & Manter 2012) | 184 | *P. xiamenensis* (Lai & Shao 2008) |
| 89 | *P. lactis* (von Neubeck *et al.* 2017) | 185 | *P. xinjiangensis* (Liu *et al.* 2009) |
| 90 | *P. libanensis* (Dabboussi *et al.* 1999) | 186 | *P. yamanorum* (Arnau *et al.* 2015) |
| 91 | *P. lini* (Delorme *et al.* 2002) | 187 | *P. zeshuii* (Feng *et al.* 2012) |
| 92 | *P. linyingensis* (He *et al.* 2015) | 188 | *P. zhaodongensis* (Zhang *et al.* 2015) |
| **reference^LPSN^** – reference according to LSPN (http://www.bacterio.net) | | | |

**Supplementary Table 6.** Phylogenomic location of type strains not considered in previous MLSA analyses

| **monophyletic group** | **newly added type strain** |
| --- | --- |
| *P. pertucinogena* | *P. abyssi* MT 5, *P. 'gallaeciensis'* V113, *P. oceani* DSM 100277 |
| *P. alcaligenes* (new group) | *P. fluvialis* ASS-1, *P. 'pharmafabricae'* ZYSR67-Z |
| *P. oleovorans* | *P. oleovorans* ssp. *lubricantis* RS1, *P. 'sediminis'* PI11 |
| *P. stutzeri* | *P. 'songnenensis'* NEAU-ST5-5, *P. zhaodongensis* NEAU-ST5-21 |
| *P. aeruginosa* | *P. furukawaii* KF707 |
| *P. putida* | *P. 'asiatica'* RYU5, *P. 'sichuanensis'* WCHPs060039, *P. 'qingdaonensis'* JJ3 |
| *P. lutea* | *P. 'bohemica'* IA19 |
| *P. syringae* | *P. floridensis* GEV388 |
| *P. chlororaphis* | *P. chlororaphis* ssp. *piscium* DSM 21509 |
| *P. jessenii* | *P. 'laurylsulfatovorans'* AP3_22 |
| *P. mandelii* | *P. silesiensis* A3 |
| *P. fragi* | *P. 'saxonica'* DSM 108989 |
| *P. fluorescens* | *P. 'haemolytica'* DSM 108987 |
| *no affiliation* | *P. hussainii* JCM 19513, *P. 'mangrovi'* TC11, *P. pohangensis* DSM 17875 |

**Supplementary Table 7.** *Pseudomonas* type strains carrying mutations in the *aprX* gene

| **type strain** | **mutation/abnormality** |
| --- | --- |
| *P. lundensis* DSM 6252 | only partially present due to end of contig |
| *P. savastanoi* ICMP 4352 | frame-shift mutation due to 4-bp deletion |
| *P. meliae* CFBP 3225 | frame-shift mutation due to 16-bp deletion |
| *P. ficuserectae* ICMP 7848 | nonsense mutation (premature stop codon) |
| *P. cannabina* ICMP 2823 | coding sequence splitted into 2 parts (5’ and 3’) due to >600-bp insertion |

**Supplementary Table 8.** Frequency of occurrence of the *aprX* locus on the level of monophyletic groups

| **monophyletic group** | **number of**  **type strains** | **frequency of type**  **strains containing *aprX*** |
| --- | --- | --- |
| *P. fluorescens* | 26 | 96.2 % |
| *P. gessardii* | 4 | 100 % |
| *P. fragi* | 10 | 50 % |
| *P. mandelii* | 6 | 100 % |
| *P. jessenii* | 5 | 20 % |
| *P. koreensis* | 4 | 100 % |
| *P. corrugata* | 4 | 100 % |
| *P. chlororaphis* | 6 | 100 % |
| *P. asplenii* | 2 | 100 % |
| *P. syringae* | 16 | 87.5 % |
| *P. lutea* | 4 | 20 % |
| *P. rhizosphaerae* | 2 | 0 % |
| *P. putida* | 20 | 30 % |
| *P. anguilliseptica* | 9 | 0 % |
| *P. straminae* | 5 | 0 % |
| *P. aeruginosa* | 17 | 5.9 % |
| *P. stutzeri* | 7 | 0 % |
| *P. oleovorans* | 11 | 0 % |
| *P. alcaligenes* | 3 | 0 % |
| *P. luteola* | 2 | 0 % |
| *P. oryzihabitans* | 2 | 0 % |
| *P. pertucinogena* | 12 | 0 % |

**Supplementary Table 9.** Abundance of *aprX-lipA2* genetic organizations at the level of species and individual strains

| ***aprX-lipA2***  **genetic composition** | **type** | **species (number of strains with genetic organization)** | | | **number**  **species** | **number**  **strains** | **cumulative**  **percentage**  **of species** |
| --- | --- | --- | --- | --- | --- | --- | --- |
| *aprXIDEF prtAB lipA2* | 1 | *P. proteolytica* (8)  *Pseudomonas* sp. 9 (4)  *P. fluorescens* (2)  *Pseudomonas* sp. 8 (2)  *P. arsenicoxydans* (1)  *P. costantinii* (1)  *P. orientalis* (1)  *P. salomonii* (1)  *P. synxantha* (1)  *P. yamanorum* (1) | *Pseudomonas* sp. 10 (5)  *P. rhodesiae* (3)  *P. ‘haemolytica’* (2)  *Pseudomonas* sp. 11 (2)  *P. azotoformans* (1)  *P. extremorientalis* (1)  *P. palleronia* (1)  P. simiae (1)  *P. tolaasii* (1) | *P. gessardii* (4)  *P. brenneri* (2)  *P. poae* (2)  *Pseudomonas* sp. 12 (2)*  *P. canadensis* (1)  *P. libanensis* (1)  *P. paralactis* (1)  *Pseudomonas* sp. 13 (1)  *P. trivialis* (1) | 28 | 54 | 32.2 |
| *aprXIDEF lipA2* | 2 | *P. fragi* (10)*  *P. deceptionensis* (1)  *P. lundensis* (1)*  *P. migulae* (1)  *P. versuta* (1) | *P. veronii* (5)  *P. grimontii* (1)  *P. lurida* (1)  *P. silesiensis* (1) | *P. panacis* (2)  *P. kilonensis* (1)  *P. marginalis* (1)  *Pseudomonas* sp. 7 (1) | 13 | 27 | 47.1 |
| *aprXIDEF \| prtAB* | 3 | *Pseudomonas* sp. 6 (3)  *P. cerasi* (1)  *P. floridensis* (1)  *P. tremae* (1) | *P. amygdali* (1)  *P. congelans* (1)  *P. graminis* (1)  *P. viridiflava* (1) | *P. caricapapayae* (1)  *P. ficuserectae* (1)  *P. syringae* (1) | 11 | 13 | 59.8 |
| *aprXIDEF prtAB lipA1A2* | 4 | *P. chlororaphis* (5)  *P. baetica* (1)  *P. lini* (1) | *P. koreensis* (2)  *P. granadensis* (1)  *P. prosekii* (1) | *P. mandelii* (2)  *P. ‘kribbensis‘* (1) | 8 | 14 | 69.0 |
| *aprXIDEF \| prtAB \| lipA2* | 5 | *P. donghuensis* (1)  *P. soli* (1) | *P. entomophila* (1)  *P. wadenswilerensis* (1) | *P. mosselii* (1) | 5 | 5 | 74.7 |
| *aprXIDEF prtB lipA2* | 6 | *P. corrugata* (1)  *P. thivervalensis* (1) | *P. mediterranea* (1) | *P. mucidolens* (1) | 4 | 4 | 79.3 |
| *aprXIDEF* | 7 | *P. cannabina* (1) | *P. cedrina* (1) | *P. meliae* (1) | 3 | 3 | 82,8 |
| *aprXIDEF lipA2 \| prtAB* | 8 | *P. lundensis* (11)* | *P. weihenstephanensis* (2) |  | 2 | 13 | 83.9 |
| *aprXIDEF prtAB lipA2 \| lipA1* | 9 | *P. lactis* (5) | *Pseudomonas* sp. 12 (1)* |  | 2 | 6 | 85.1 |
| *aprXIDEF \| prtA* | 10 | *P. avellanae* (1) | *P. savastanoi* (1) |  | 2 | 2 | 87.4 |
| *aprXH1H2IDEF lipA2*** | 11 | *P. asplenii* (1) | *P. fuscovaginae* (1) |  | 2 | 2 | 89.7 |
| *aprXIDEF lipA1 prtAB lipA2* | 12 | *P. protegens* (3) |  |  | 1 | 3 | 90.8 |
| *aprXI \| aprDEF lipA2* | 13 | *P. fragi* (3)* |  |  | 1 | 3 | 90.8 |
| *aprDEFXI \| prtA* | 14 | *P. aeruginosa (2)* |  |  | 1 | 2 | 92.0 |
| *aprXIDEF \| lipA2* | 15 | *P. ‘sichuanensis’* (1) |  |  | 1 | 1 | 93.1 |
| *aprXIDEF lipA1 prtB lipA2* | 16 | *Pseudomonas* sp. 5 (1) |  |  | 1 | 1 | 94.3 |
| *aprXIDEF prtA lipA2* | 17 | *P. antarctica* (1) |  |  | 1 | 1 | 95.4 |
| *aprXIDEF prtB lipA1A2* | 18 | *P. jessenii* (1) |  |  | 1 | 1 | 96.6 |
| *aprX* | 19 | *P. saponiphila* (1) |  |  | 1 | 1 | 97.7 |
| *aprXI \| aprDEF* | 20 | *P. ‘massiliensis’* (1) |  |  | 1 | 1 | 98.9 |
| *aprXID \| aprDEF* | 21 | *P. fragi* (1)* |  |  | 1 | 1 | 98.9 |
| *aprXI \| aprD \| aprEF* | 22 | *P. asturiensis* (1) |  |  | 1 | 1 | 100.0 |
| * Indicates species with strains having different *aprX-lipA2* genetic organizations  ** Genetic organization in which two hypothetical protease genes (named apr*H1* and apr*H2*) are located between *aprX* and *aprI*  *\|* Indicates that gene clusters are located at different genomic positions | | | | | | | |
